# A comparative study of multi-omics integration tools for cancer driver gene identification and tumour subtyping

**DOI:** 10.1093/bib/bbz121

**Published:** 2019-11-27

**Authors:** Anita Sathyanarayanan, Rohit Gupta, Erik W Thompson, Dale R Nyholt, Denis C Bauer, Shivashankar H Nagaraj

**Affiliations:** 1 School of Biomedical Sciences, Faculty of Health, and Institute of Health and Biomedical Innovation, Queensland University of Technology, Brisbane, Australia; 2 Department of Biotechnology, Indian Institute of Technology Madras, Chennai, India; 3 Department of Computational Biology, Indraprastha Institute of Information Technology, Delhi, India; 4 Translational Research Institute, Brisbane, Australia; 5 CSIRO, Health & Biosecurity, Sydney, Australia

**Keywords:** multi-omics data, cancer, multi-staged integration, meta-dimensional integration, tools evaluation

## Abstract

Oncogenesis and cancer can arise as a consequence of a wide range of genomic aberrations including mutations, copy number alterations, expression changes and epigenetic modifications encompassing multiple omics layers. Integrating genomic, transcriptomic, proteomic and epigenomic datasets via multi-omics analysis provides the opportunity to derive a deeper and holistic understanding of the development and progression of cancer. There are two primary approaches to integrating multi-omics data: multi-staged (focused on identifying genes driving cancer) and meta-dimensional (focused on establishing clinically relevant tumour or sample classifications). A number of ready-to-use bioinformatics tools are available to perform both multi-staged and meta-dimensional integration of multi-omics data. In this study, we compared nine different integration tools using real and simulated cancer datasets. The performance of the multi-staged integration tools were assessed at the gene, function and pathway levels, while meta-dimensional integration tools were assessed based on the sample classification performance. Additionally, we discuss the influence of factors such as data representation, sample size, signal and noise on multi-omics data integration. Our results provide current and much needed guidance regarding selection and use of the most appropriate and best performing multi-omics integration tools.

## Introduction

Alterations in the genetic make-up of a cell, such as mutations, copy number aberrations and epigenetic changes, can all drive the development of cancer [1]. Copy number aberrations, which involve the gain or loss of genomic DNA, contribute to oncogenesis and tumour progression by activating oncogenes or inactivating tumour suppressor genes [2]. Epigenetic mechanisms including methylation of cytosine bases play crucial roles in regulating gene expression during normal mammalian development. However, disruption of such regulatory mechanisms can cause hypermethylation or hypomethylation of gene promoter regions and can lead to silencing of critical tumour suppressor functions [3].

Despite these well-understood mechanisms, two major challenges remain for the studies of oncogenesis. The first challenge is the identification of genes and aberrations that serve as drivers for disease development, which is complicated by the evolutionary nature of the disease and the presence of passenger aberrations that do not contribute to disease development [4]. Identification of such genetic modulating factors can shed vital light on the underlying molecular mechanisms of disease. The second challenge is the stratification of samples or patients to discover clinically relevant molecular subtypes or treatment groups that can aid in improved patient prognosis and treatments. Conventional approaches to identify driver genes involve performing differential analyses using individual ‘omics’ datasets (such as identification of differentially expressed genes or methylated regions) [5], while approaches to identify relevant patient or disease subtypes involve concatenation of the individual omics datasets followed by classification using techniques such as Random Forests [6] or clustering of clusters obtained from individual omics datasets [7]. However, such analyses fail to provide insight into the interactions between the different regulatory systems encapsulated by different omics data types.

Multi-omics data integration allows the joint analysis of multiple omics data types to provide a global view of the biological system and offers insights into the nature of the interactions between the different dataset layers. As cancer is a heterogeneous genetic disease, it is imperative that such multi-omics data integrations be performed in order to fully appreciate the complex inter-layer regulatory interactions governing the development and progression of this disease. Decreasing costs and technological advancement have enabled the multi-platform sequencing of thousands of tumours by consortia such as International Cancer Genome Consortium [8] and The Cancer Genome Atlas (TCGA) [9], driving a shift towards integrative multi-omics investigations of cancer.

Multi-omics integration strategies commonly employed to address the aforesaid challenges can broadly be classified into two types: multi-staged and meta-dimensional integrative approaches [10]. Multi-staged integration (also known as linear integration or sequential integration) is based on the linear hypothesis that variations in DNA lead to changes in gene expression levels, which in turn cause protein expression changes leading to diseases or phenotypic changes [11]. This approach models the relationship between two given omics data types as linear and hierarchical. The objective of this approach is to uncover cause–effect links (*cis* relationships), such as the effects of copy number aberration on the expression of the affected gene. Tools that employ multi-staged integration approaches perform gene-centric integration in order to uncover the potential driver genes and help understand the effects of the interplay between different gene regulatory levels on oncogenesis and cancer progression. There are numerous ready-to-use tools available which employ this approach, including CNAmet [12], iGC [13], PLRS [14], Oncodrive-CIS [15] and MethylMix [16].

Meta-dimensional integration (or simultaneous integration) is based on the hypothesis that interactions between the multiple biomolecular layers are non-hierarchical and complex [17]. This approach involves the simultaneous fusion of information derived from two or more omics data types and is commonly applied for problems such as tumour subtype discovery [18–20], biomarker identification [21] and the exploration of perturbed signalling pathways [22]. The integration is performed at sample level, and commonly used tools include SNF [23], BCC [24], iClusterPlus [25] and mixOmics [26].

Given the increasing availability of multi-omics datasets and the continuous development of analytical approaches and objectives, novel multi-omics integration tools are continuously being developed, and selection of the most appropriate and best performing integration tool from the numerous tools available can be a tedious and time-consuming task. Although previous comparative studies have investigated the performance of multi-omics integration tools, they have focused only on either the multi-staged or the meta-dimensional approaches and often failed to investigate the effects of gene (or feature) selection [27], influence of dataset size [28] and multi-class classification [29]. To bridge these knowledge gaps, in this article we present an overview of both the multi-staged and meta-dimensional integration tools currently available and conduct a systematic evaluation of these tools based on their performance. The multi-staged integration tools were evaluated based on their ability to identify cancer-associated genes, functional Gene Ontology (GO) terms and relevant pathways, while the meta-dimensional integration tools were evaluated based on their sample classification performance. Overall, our study is intended to aid users in the selection of tools for multi-omics integrative analyses.

## Methods

Five multi-staged (CNAmet, iGC, PLRS, Oncodrive-CIS and MethylMix) and four meta-dimensional (SNF, BCC, iClusterPlus and mixOmics) tools were compared in this study and are summarized in the following sections and in Tables 1 and 2, respectively.

**Table 1:** Summary of multi-staged integration tools used in this study

Tool	Omics data	Input	Output	Method	Reference
CNAmet	CN, ME and GE	Gene-level CN and/or ME as binary matrices and GE matrix	Weights, scores and associated *P*-values, and FDR for each gene	Independent association of the omics using signal-to-noise ratio weights followed by combining weights to obtain a score	[12]
iGC	CN and GE	Gene-level segmented or thresholded CN and GE matrices	List of genes for which expression is driven by amplification and deletion with associated *P*- and FDR values	Student’s *t*-test with unequal variance	[13]
PLRS	CN and GE	Gene-level GE, CN, thresholded CN with CN call probabilities (optional) matrices	Spline coefficients of the model fitted for the genes, *P*-values and FDR values	Piecewise linear regression splines	[14]
Oncodrive-CIS	CN and GE	Gene-level GE matrix and thresholded CN	Scores for each gene that represent the bias towards expression dysregulation due to copy number change	Estimation of impact of CN change on GE followed by estimation of standard scores of the impact in tumour and normal samples and finally combining the standard scores	[15]
MethylMix	ME and GE	Probe-level or gene-level ME and gene-level GE matrices	List of transcriptionally predictive and differentially methylated genes	Linear regression followed by modelling using beta mixture model	[16]

CN, copy number; GE, gene expression; ME, methylation.

**Table 2:** Summary of meta-dimensional integration tools used in this study

Tool	Omics data	Input	Output	Method	Reference
SNF	Any omics	Matrices of the omics data	Cluster assignments of samples	Weighted similarity network fusion	[23]
BCC	GE, ME, miRNA, proteomics	Matrices of the omics data	Adherence values and cluster assignments of samples for multi-omics clustering and individual omics clustering	Bayesian clustering based on Dirichlet mixture model	[24]
iClusterPlus	Any omics	Matrices of the omics data	Cluster assignments of samples and multi-omics gene signature	Joint modelling followed by feature selection using lasso	[25]
mixOmics	Any omics	Matrices of the omics data and the labels associated with samples	Prediction of class labels for test data and multi-omics gene signature	Sparse generalized canonical correlation analysis. Feature selection using lasso	[26]

GE, gene expression; ME, methylation.

### Multi-staged integration tools

This study assessed the performance of the CNAmet, iGC, PLRS, Oncodrive-CIS and MethylMix tools designed to carry out the multi-staged integration. A generalized workflow implemented in these tools to achieve integration and identify driver genes involves quantification of the strength of the cause and effect association between aberration in the DNA (copy number and methylation aberration) and mRNA expression at the gene level, using a statistical approach followed by correction for multiple hypothesis testing in order to identify the statistically significant genes. The statistical approaches include variations of linear regression techniques [30], Student’s *t*-tests [13] and correlation tests [16].

While iGC, PLRS and Oncodrive-CIS tools attempt to discover the genes that have dysregulated expression due to copy number change by integrating copy number and gene expression data, the MethylMix tool integrates methylation and gene expression data to identify the genes for which expression is influenced by methylation status. The CNAmet tool is capable of modelling both the combined as well as independent effects of a given gene’s copy number and methylation status on its expression. In this study, we used the CNAmet tool to identify the independent effects of the copy number and methylation aberrations on the gene expression. Table 1 summarizes the details regarding the reviewed multi-staged integration tools (see Supplementary Data section for more details).

### Meta-dimensional integration tools

The meta-dimensional integration tools assessed in this study include the SNF, BCC, iClusterPlus and mixOmics. These tools employ one or more mathematical techniques including matrix factorization, correlation, Bayesian, network and transformation [31, 32], for the simultaneous integration of multiple omics data types.

The SNF tool integrates multi-omics data using transformation-based network fusions. Briefly, each omics data type is transformed into a network wherein the nodes represent samples and weighted edges represent the similarity between the samples. Next, these individual networks are fused into a single network using message-passing theory method [33], where high similarity edges between the samples in one or more omics data types are retained, and inconsistent low similarity edges are removed. Finally, the subtypes are estimated using a spectral clustering method. However, the SNF tool does not provide the users with the information on the important features responsible for the observed subtypes. The BCC tool performs Bayesian consensus clustering based on an extended Dirichlet mixture model for multiple omics data types. This method assumes that there is a separate clustering observed in each omics data type and that these clusters adhere loosely to an overall clustering. Although this method provides information regarding similarity between clusters of independent omics data types and the consensus clustering through adherence parameter (α), this method does not convey the important genes associated with the clustering. The iClusterPlus tool performs model-based matrix factorization integration. Here, each omics data type is decomposed into a components factor and a loading factor based on different modelling assumptions for different omics data types. The components factor represents the common latent cancer subtypes, and the loading factor represents the gene features. The important features are selected using lasso regularization [34], and the clusters are identified using a K-means clustering algorithm. The mixOmics tool also employs a matrix factorization-based method, which extends the sparse generalized canonical correlation analysis to a supervised classification problem. Here, the correlation between the components factors is maximized and projected to a smaller dimensional space, and the important features are selected by lasso regularization [34]. The SNF, BCC and iClusterPlus tools perform an unsupervised classification of the integrated multi-omics data that are effective for data exploration and discovery of novel subtypes, while the mixOmics tool performs supervised classification in which the sample subtype is also provided as an input during integration. Table 2 summarizes the details on these meta-dimensional tools (see Supplementary Data section for more details).

### Datasets used for multi-staged integration tool analyses

Level 3 TCGA data pertaining to four cancers, mesothelioma [35], colon [36], pancreatic [37] and melanoma [38], processed and hosted by the UCSC Xena [39] and the firebrowse (http://firebrowse.org/) databases, were used in this study. These cancers were selected as they have varying sample size and are widely studied. The following omics data were downloaded for each cancer (refer to Supplementary Table S1 for more details):

- Copy number variation: Gene-level GISTIC 2.0 [40] segmented and thresholded copy number variation downloaded from the UCSC Xena database. While the segmented data contain the copy number estimates, the thresholded copy number data contain the thresholded estimates, i.e. −2, −1, 0, 1 and 2, representing homozygous deletion, single copy deletion, diploid normal copy, low-level copy number amplification and high-level copy number amplification, respectively.
- Gene expression: Gene-level transcription estimates, as in log_2_(x+1) transformed RSEM [41] normalized expression counts downloaded from the UCSC Xena database.
- DNA methylation: Gene-level mean beta values were downloaded from the firebrowse database.

**Figure 1:**
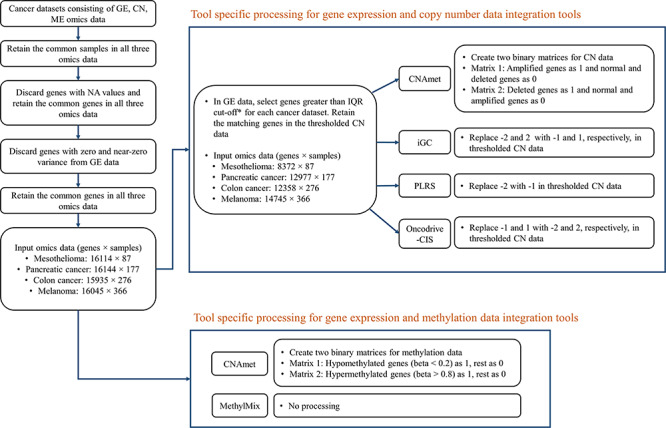
Scheme outlining the data processing steps for multi-staged tools.

Data processing for each cancer multi-omics dataset involved selection of tumour samples common across all omics, removal of genes with NA, zero variance and near-zero variance (i.e. genes with unique values in the samples is less than 10% and the ratio of the frequency of the most common value to the frequency of the second most common value is greater than 95:5) from gene expression omics data using caret R package [42] followed by selection of common genes across all omics. When integrating gene expression and copy number data, the presence of genes with small inter-quartile range (IQR) of expression caused a runtime error in the Oncodrive-CIS tool. Therefore, the smallest permissible IQR threshold was identified for each cancer dataset by deleting in a listwise manner until the tool ran without error. The IQR thresholds for mesothelioma, pancreatic cancer, colon cancer and melanoma datasets were 0.90, 0.50, 0.55 and 0.55, respectively. These final datasets were used for all tools integrating copy number and gene expression to perform an unbiased comparison. Further tool-specific processing is outlined in Figure 1.

### Datasets used for meta-dimensional integration tool analyses

Meta-dimensional tool analyses were performed using 2 real multi-omics cancer datasets and 16 simulated datasets.

#### Real datasets

Hepatocellular carcinoma (HPC) dataset: This dataset [43] comprises the sequencing data for primary tumour (Tumour), adjacent normal tissue (Normal) and portal vein tumour thrombosis (PVTT) samples from 20 HPC patients, totalling 60 samples. The mRNA gene expression (GSE77509; measured using Illumina HiSeq 2500), miRNA gene expression (GSE76903; measured using Illumina HiSeq 2500) and probe-level methylation (GSE77269; measured using Illumina HumanMethylation450 BeadChip) data were downloaded from Gene Expression Omnibus database [44]. Quality control included log transformation of miRNA and mRNA expression data and removal of genes or probes with null values in more than 25% of samples. We used inter-sample variance to select the most informative features [45]. The top 10% of the variable mRNA and miRNA gene expression and top 5% of methylation probes were chosen for integration analysis as these features captured most of the variance in the dataset (Supplementary Table S6 and Supplementary Figure S6).

Glioblastoma (GBM) dataset: Protein expression measured using reverse phase protein array technology, gene expression measured using AffyU133a array, DNA methylation data measured using Illumina Infinium HumanMethylation27 platform and phenotype information for glioblastoma tumour samples were downloaded from UCSC Xena database. Common samples across all omics data and having information on the clinical subtype were selected for analysis. Feature selection was based on inter-sample variance, with the top 25% variable genes and probes selected (Supplementary Table S6 and Supplementary Figure S7). The final dataset contained 100 samples belonging to 4 clinical subtypes: Classical (25 samples), Mesenchymal (27 samples), Neural (17 samples) and Proneural (31 samples).

#### Simulated datasets

The miRNA gene expression data, DNA methylation data and the top 1000 inter-sample variable genes from mRNA gene expression data from the TCGA breast cancer dataset [46] (Supplementary Table S7) were used to create simulated datasets using a previously detailed method [28].

Briefly, four simulated datasets A, B, C and D were created. Each simulated dataset contained three matrices referred to as Omics data 1, Omics data 2 and Omics data 3, simulated with rows corresponding to 60 samples and columns corresponding to 500 features. In each simulated matrix, the samples were modelled using Gaussian distributions such that they were equally distributed among three classes (i.e. subtypes). Additionally, independent Gaussian noise (mean = 0, standard deviation = 0.4) was added to the features profiles. The profiles of the features in the samples belonging to each class with added independent noise are given below: Class 1 − *Normal (m_i_,sd_i_) + Normal(0,0.4)* Class 2 − *Normal (m_i_ – m_i_/2, sd_i_) + Normal(0,0.4)* Class 3 − *Normal (m_i_ + m_i_/2, sd_i_ + sd_i_/10) + Normal(0,0.4)* where *m_i_* and *sd_i_* are the mean and standard deviation, respectively, calculated from 500 randomly selected features from the real omics data types.

To assess the ability of the tools to combine the patterns identified in individual omics data types, certain classes in two or all of the omics data types of the simulated datasets B, C and D were simulated to be a hidden class (Table 3). Simulated dataset A contained no hidden class. A hidden class contained samples whose feature profiles were similar to that of the samples in the remaining two classes. Presence of such classes in datasets reduces the signal strength thereby making the dataset complex.

**Table 3:** Details of the classes that were simulated as hidden class in different omics data in each simulated dataset

Simulated datasets	Omics data 1	Omics data 2	Omics data 3
A	No hidden classes		
B	Class 3	Class 1	No hidden class
C	Class 3	Class 2	Class 2
D	Class 3	Class 2	Class 1

To understand the effect of sample size on the performance of these tools, all the datasets were recreated with 150 samples, with each class containing 50 samples. The 150-sample datasets will be referred to as Large Group, while the previously created 60-sample datasets will be referred to as Small Group. To assess the influence of noise, Gaussian noisy features with means equal to that of the genes selected randomly from the real dataset and with a standard deviation of 2 were added to the datasets in both the Small and Large Groups and are referred to as Small Noisy Group and Large Noisy Group, respectively. As these (noisy) features have a constant standard deviation across all three classes, they do not have the information to discriminate the classes and hence, are noisy. For all the datasets, 100 noisy features were added to Omics data 1, while 20 noisy features were added to Omics data 2 and 3.

### Running the multi-omics integration tools

The CNAmet, iGC, PLRS, MethylMix, SNF and BCC tools were run in R on a 64-bit Ubuntu desktop computer. The iClusterPlus and mixOmics tools were also run in R but on a high performance computing Linux cluster hosted by Queensland University of Technology, Brisbane, Australia. The Oncodrive-CIS tool, which is available as a Python script, was executed via the terminal (command line) in Python version 2.7 on a 64-bit Ubuntu desktop. All the tools were run using their default parameter settings obtained from the respective manuals whereas the parameter values for the mixOmics tool were obtained from their case study (http://mixomics.org/mixdiablo/case-study-tcga/). The runtimes of these tools were recorded as the time taken to execute the integration function in each tool, and the data pre-processing time prior to running these tools was not included. We have provided the R code to reproduce the pre-processing, variable selection, creation of simulated datasets, running of tools and performance evaluation presented in this study on GitHub (https://github.com/AtinaSat/Evaluation-of-integration-tools).

### Performance evaluation of multi-staged integration tools

Due to differences in the omics data types integrated by the multi-staged tools, separate evaluations were conducted for the tools integrating copy number and gene expression data (Oncodrive-CIS, iGC, PLRS and CNAmet tools) and the tools integrating methylation intensity and gene expression data (MethylMix and CNAmet tools).

The tools were evaluated based on the overlap estimates of genes, function terms (GO terms) and pathways with that of gold standard cancer gene list—cBioPortal cancer gene list (CCGL; http://www.cbioportal.org/cancer_gene_list.jsp) [47] which contains 981 known cancer genes. To evaluate the reliable predictions from the tools as well as not miss the identified cancer genes, a maximum of 1500 genes driven by amplification (or hypomethylation) and a maximum of 1500 genes driven by deletion (or hypermethylation) with FDR values <0.05 were retained for each tool. The two lists were combined, and the unique genes were identified for performance assessment. As the PLRS tool does not provide the distinction between amplification and deletion among the results, to ensure tool evaluations were based on datasets consistent in size, a maximum of 3000 genes with FDR values <0.05 were selected for evaluation. Similarly, the MethylMix tool does not provide the distinction between hypermethylation and hypomethylation among the results; hence, a maximum of 3000 genes with FDR values <0.05 were selected for evaluation.

**Figure 2:**
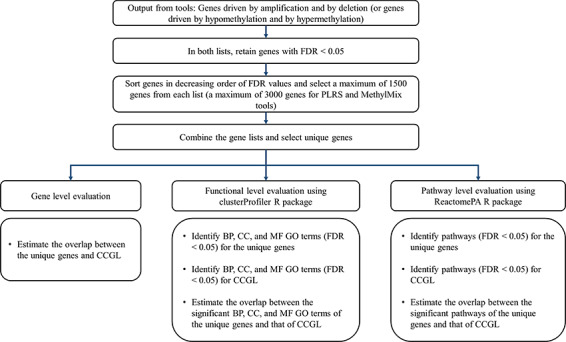
Scheme for selection of significant genes from the results and evaluation of multi-staged tools.

The selected gene result from each tool and the cancer gene list were enriched with GO terms for Biological Process (BP), Cellular Component (CC) and Molecular Function (MF) categories using clusterProfiler R package [48] and the Reactome pathways [49] using the ReactomePA R package [50]. While the gene-level evaluation is based on estimating the number of overlapping genes, the function and pathway level evaluations are based on estimating the number of overlapping enriched GO function terms and Reactome pathways, respectively, between the results and the cancer gene list. Figure 2 outlines the steps for the selection of genes and evaluation at the gene, function and pathway levels.

### Performance evaluation of meta-dimensional integration tools

The meta-dimensional tools were evaluated based on the sample classification performance in various scenarios: (i) binary and multi-class classification, (ii) presence of hidden classes, (iii) small and large sample sizes and (iv) noise in small and large sample sizes. The HPC dataset was used for the binary classification analysis in which the sample classes were Normal and PVTT/Tumour, with the latter class formed by merging the PVTT and Tumour samples. For multi-class classification analysis, the HPC and GBM datasets were used. The sample classes for the HPC dataset were Normal, PVTT and Tumour, while for the GBM dataset the classes were Classical, Mesenchymal, Neural and Proneural. As the mixOmics tool performs supervised classification, the samples in both the real and simulated datasets were divided into training (75%) and test (25%) datasets prior to analysis. The tool performance was assessed using the classification results obtained for the test datasets.

The sample classification powers of the meta-dimensional integration tools were assessed using the following metrics:

$$\mathrm{Precision}=\frac{\mathrm{True}\ \mathrm{positives}}{\mathrm{True}\ \mathrm{positives}+\mathrm{False}\ \mathrm{positives}\ }$$

$$\mathrm{Recall}=\frac{\mathrm{True}\ \mathrm{positives}}{\mathrm{True}\ \mathrm{positives}+\mathrm{False}\ \mathrm{negatives}\ }$$

$${\mathrm{F}}_1=\frac{2\ast \mathrm{Precision}\ast \mathrm{Recall}}{\mathrm{Precision}+\mathrm{Recall}\ }$$

$$\mathrm{Avg}.{\mathrm{F}}_1=\frac{\sum_{\mathrm{k}=1}^{\mathrm{n}}{{\mathrm{F}}_1}_{\mathrm{k}}}{\mathrm{n}}\ \left(\mathrm{n}=\mathrm{number}\ \mathrm{of}\ \mathrm{classes}\right)$$

$$\mathrm{Accuracy}=\frac{\mathrm{True}\ \mathrm{positives}}{\mathrm{Total}\ \mathrm{number}\ \mathrm{of}\ \mathrm{samples}\ }$$

In the following section, we have highlighted the performance of meta-dimensional tools mainly based on their average F_1_ scores and accuracies. Refer to Supplementary Tables S9–S12 for precision and recall scores achieved by these tools for the simulated datasets.

## Results

### Multi-staged integration tools analyses

#### Copy number and gene expression integration

The performance of the copy number and gene expression integration tools in the mesothelioma, pancreatic cancer, colon cancer and melanoma datasets were tested at three evaluation levels: gene, function and pathway. The 3000 most informative and statistically significant unique genes identified by these tools were utilized for the evaluations. For these gene lists and the CCGL, significant GO function terms and pathways were identified. For each evaluation, overlaps of the results from the tools with the CCGL in each dataset were identified and are shown in Table 4. A strong overlap demonstrates high sensitivity of the tool to identify cancer-associated genes, GO function terms and pathways. A total of 20 overlap tests were performed (1 gene, 3 functions and 1 pathway for each of the 4 datasets) for each tool.

**Table 4:** Gene, function and pathway level comparisons between multi-staged tools integrating copy number variation and gene expression data and the CCGL. The numbers in parentheses in the heading indicate the number of features identified using CCGL in each category. The numbers in parentheses in the results indicate the percentage of overlapping features with respect to CCGL features. The numbers in bold indicate the highest overlap in each category in a cancer dataset

Dataset	Tools	Genes		Functional—BP GO terms		Functional—CC GO terms		Functional—MF GO terms		Pathways		Total number of overlapping features
		Top significant genes	Overlap with CCGL (981)	BP terms for top significant genes	Overlap with CCGL (2649)	CC terms for top significant genes	Overlap with CCGL (118)	MF terms for top significant genes	Overlap with CCGL (232)	Pathways for top significant genes	Overlap with CCGL (462)	
Mesothelioma	CNAmet	1997	103 (10.50)	0	0 (0.00)	6	0 (0.00)	0	0 (0.00)	0	0 (0.00)	103
	iGC	1155	63 (6.42)	0	0 (0.00)	5	0 (0.00)	4	0 (0.00)	0	0 (0.00)	63
	PLRS	2472	**124 (12.64)**	6	**1 (0.04)**	19	**7 (5.93)**	4	**3 (1.29)**	1	0 (0.00)	**135**
	Oncodrive-CIS	663	43 (4.38)	0	0 (0.00)	0	0 (0.00)	0	0 (0.00)	0	0 (0.00)	43
Pancreatic cancer	CNAmet	2824	147 (14.98)	17	11 (0.42)	12	0 (0.00)	15	4 (1.72)	7	0 (0.00)	162
	iGC	2495	138 (14.07)	34	9 (0.34)	11	5 (4.24)	6	2 (0.86)	3	0 (0.00)	154
	PLRS	3000	**180 (18.35)**	70	27 (1.02)	19	3 (2.54)	32	13 (5.60)	10	5 (1.08)	**228**
	Oncodrive-CIS	2150	122 (12.44)	86	**37 (1.40)**	45	**7 (5.93)**	35	**14 (6.03)**	30	**10 (2.16)**	190
Colon cancer	CNAmet	2539	146 (14.88)	91	64 (2.42)	3	0 (0.00)	2	0 (0.00)	6	5 (1.08)	215
	iGC	2879	181 (18.45)	256	144 (5.44)	92	22 (18.64)	41	12 (5.17)	127	32 (6.93)	391
	PLRS	3000	**188 (19.16)**	424	**224 (8.46)**	135	**27 (22.88)**	79	**23 (9.91)**	187	**47 (10.17)**	**509**
	Oncodrive-CIS	2733	165 (16.82)	273	142 (5.36)	120	26 (22.03)	73	18 (7.76)	107	30 (6.49)	381
Melanoma	CNAmet	2366	140 (14.27)	156	131 (4.95)	43	18 (15.25)	7	1 (0.43)	15	9 (1.95)	299
	iGC	2876	172 (17.53)	669	**306 (11.55)**	249	42 (35.59)	164	46 (19.83)	393	**86 (18.61)**	652
	PLRS	3000	**198 (20.18)**	472	224 (8.46)	224	43 (36.44)	147	42 (18.10)	310	57 (12.34)	564
	Oncodrive-CIS	2758	181 (18.45)	653	302 (11.40)	241	**48 (40.68)**	158	**52 (22.41)**	356	81 (17.53)	**664**

The PLRS tool achieved the highest overlap with CCGL in 11/20 overlap tests, demonstrating the highest sensitivity compared to other tools. The Oncodrive-CIS and iGC tools achieved the highest overlap in 6/20 and 2/20 tests, respectively. All tools displayed poor performance for the mesothelioma dataset. The comparatively small sample size of the mesothelioma dataset (87 samples) likely reduced the power to identify significant genes and subsequently the significant GO function terms and pathways associated with the disease. Overall, all tools showed an increasing trend in the performance with increasing sample size of the datasets.

**Figure 3:**
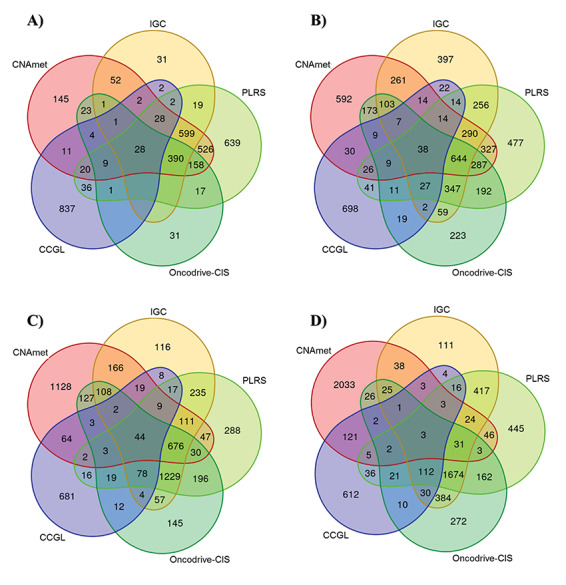
Congruence between the significant genes identified by CNAmet, iGC, PLRS, Oncodrive-CIS tools (copy number and gene expression integrating tools) and the CCGL genes in the (**A**) mesothelioma, (**B**) pancreatic cancer, (**C**) colon cancer and (**D**) mesothelioma datasets.

We compared the approaches implemented by these tools using Venn diagrams of the results at all evaluation levels for all datasets. High congruence was observed between the iGC, PLRS and Oncodrive-CIS tools at gene level in the pancreatic cancer (35–49%), colon cancer (67–74%) and melanoma (60–66%) datasets (Figure 3). The goal of all tools evaluated is to identify a statistically significant effect of copy number change on gene expression. The iGC tool uses Student’s *t*-test, PLRS uses piecewise linear regression splines and Oncodrive-CIS uses Z-test. The high similarity between these statistical methods resulted in the observed high overlap of implicated genes. However, in general highest congruence was observed between PLRS and iGC tools (Supplementary Figures S1–S4).

#### Methylation and gene expression integration

**Figure 4:**
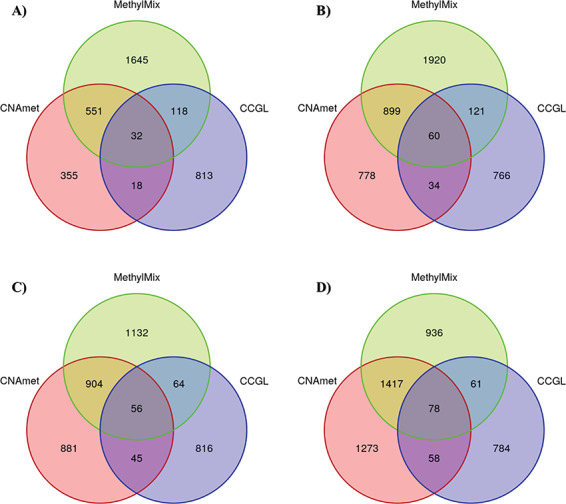
Congruence between the significant genes identified by CNAmet, MethylMix tools (methylation and gene expression integrating tools), and the CCGL genes in the (**A**) mesothelioma, (**B**) pancreatic cancer, (**C**) colon cancer and (**D**) melanoma datasets.

Similar to copy number and gene expression integration tools, methylation and gene expression integration tools were also assessed at three levels—gene, function and pathway. The top 3000 methylation-driven genes were selected from the results provided by the MethylMix and CNAmet tools to perform 20 overlap tests with CCGL. The MethylMix tool, which integrates the omics datasets using a continuous data and linear regression approach, outperformed the CNAmet tool, which integrates using a categorical data and signal-to-noise ratio approach, in 18/20 overlap tests (Table 5). The sharp cut-offs used by CNAmet tool to signify hypermethylated and hypomethylated genes could have led to the misinterpretation of the underlying relationship between methylation intensity and expression of a gene. Venn diagrams of the gene-level results between the tools showed CNAmet genes overlapped on an average of 54.73% with MethylMix genes (Figure 4). Venn diagrams of BP, CC, MF and pathway terms between the tools showed CNAmet results overlapped on an average of 67.29%, 17.85%, 43.23% and 6.88%, respectively, with MethylMix results (Supplementary Figure S5).

**Table 5:** Gene, function and pathway level comparisons between multi-staged tools integrating methylation and gene expression data and the CCGL. The numbers in parentheses in the heading indicate the number of features identified using CCGL in each category. The numbers in parentheses in the results indicate the percentage of overlapping features with respect to CCGL features. The numbers in bold indicate the highest overlap in each category in a cancer dataset

Dataset	Tools	Genes		Functional—BP GO terms		Functional—CC GO terms		Functional—MF GO terms		Pathways		Total number of overlapping features
		Top significant genes	Overlap with CCGL (981)	BP terms for top significant genes	Overlap with CCGL (2649)	CC terms for top significant genes	Overlap with CCGL (118)	MF terms for top significant genes	Overlap with CCGL (232)	Pathways for top significant genes	Overlap with CCGL (462)	
Mesothelioma	CNAmet	956	50 (5.10)	103	75 (2.83)	0	0 (0.00)	6	1 (0.43)	0	0 (0.00)	126
	MethylMix	2346	**150 (15.29)**	505	**424 (16.01)**	42	**11 (9.32)**	26	**10 (4.31)**	12	**5 (1.08)**	**600**
Pancreatic cancer	CNAmet	1771	94 (9.58)	40	31 (1.17)	0	0 (0.00)	5	3 (1.29)	0	0 (0.00)	128
	MethylMix	3000	**181 (18.45)**	614	**425 (16.04)**	31	**6 (5.08)**	13	**6 (2.59)**	19	**7 (1.52)**	**625**
Colon cancer	CNAmet	1886	101 (10.30)	21	13 (0.49)	0	0 (0.00)	2	2 (0.86)	41	**31 (6.71)**	147
	MethylMix	2156	**120 (12.23)**	95	**51 (1.93)**	16	**2 (1.69)**	11	**3 (1.29)**	3	0 (0.00)	**176**
Melanoma	CNAmet	2826	136 (13.86)	320	207 (7.81)	28	10 (8.47)	16	3 (1.29)	53	**33 (7.14)**	389
	MethylMix	2492	**139 (14.17)**	566	**429 (16.19)**	63	**14 (11.86)**	54	**19 (8.19)**	45	19 (4.11)	**620**

### Meta-dimensional integration tools analyses

#### Binary and multi-class classification of HPC dataset

The goal of binary and multi-class classification using meta-dimensional integration is to accurately identify the different tumour subtypes or patient types using multi-omics datasets. Binary classification involves segregating the samples into two subtypes/classes, while multi-class classification involves identifying more than two classes. Tools achieving high accuracies and F_1_ scores in multi-class classification are preferable as tissue-specific tumours often comprise more than two molecular subtypes that may differ in prognosis and treatment strategies.

The performances of the tools during binary and multi-class classification tested using the HPC and GBM datasets is shown in Table 6. In binary class classification, all tools performed with accuracy >0.9 except for iClusterPlus tool (0.78). The mixOmics tool outperformed the others in multi-class classification analyses by providing the highest average F_1_ scores and accuracies among all the tools. The performance of all the tools was significantly better in the binary classification compared to the multi-class classification analyses. In the former analysis, all the tools were capable of retrieving the two classes with high precision and recall. However, during the multi-class classification using the HPC dataset, only the mixOmics tool could delineate between the samples from Tumour and PVTT classes. The poor precision and recall in classifying between these two classes could have been due to the high heterogeneity in these classes, as reported by Yang *et al*. [37]. In the GBM dataset, all tools achieved the lowest performance for the Neural subtype compared to the other subtypes. The poor classification of this subtype could be due to the fact that it lacks characteristic gene abnormalities [51] caused likely by normal tissue contamination [52]. Hence, this subtype could be non-tumour-specific phenotype [53]. Multi-class classification of HPC and GBM datasets using the mixOmics tool produced better classification. The prior learning using training data enabled the tool to distinguish better the signals from the different classes, thereby resulting in superior precision and recall scores for all classes. The SNF tool achieved the second best classification in the multi-class classification scenario.

**Table 6:** Binary and multi-class classification results by meta-dimensional integration tools for HPC and GBM datasets. The numbers in parentheses beside each class indicate the number of samples belonging to the class in the cluster/total number of samples in the cluster

**Dataset**	**Metrics/Tools**	**SNF**				**BCC**				**mixOmics**				**iClusterPlus**			
HPC—Binary classification	Classes	Normal (20/23)		PVTT/Tumour (37/37)		Normal (20/24)		PVTT/Tumour (36/36)		Normal (6/7)		PVTT/Tumour (8/8)		Normal (20/33)		PVTT/Tumour (27/27)	
	Precision	0.87		1		0.83		1		0.86		1		0.61		1	
	Recall	1		0.92		1		0.9		1		0.89		1		0.68	
	F1	0.93		0.96		0.91		0.95		0.92		0.94		0.76		0.81	
	Avg F1	0.94				0.93				0.93				0.78			
	Accuracy	0.95				0.93				0.93				0.78			
HPC—Multi-class classification	Classes	Normal (20/22)	PVTT (10/19)	Tumour (10/19)		Normal (20/22)	PVTT (6/12)	Tumour (13/26)		Normal (6/7)	PVTT (3/4)	Tumour (3/4)		Normal (19/24)	PVTT (9/18)	Tumour (9/18)	
	Precision	0.91	0.53	0.53		0.91	0.5	0.5		0.86	0.75	0.75		0.79	0.5	0.5	
	Recall	1	0.5	0.5		1	0.3	0.65		1	0.75	0.6		0.95	0.45	0.45	
	F1	0.95	0.51	0.51		0.95	0.37	0.57		0.92	0.75	0.67		0.86	0.47	0.47	
	Avg F1	0.66				0.63				0.78				0.6			
	Accuracy	0.67				0.65				0.8				0.61			
GBM—Multi-class classification	Classes	Classical (23/33)	Mesenchymal (17/31)	Neural (0/10)	Proneural (23/26)	Classical (24/37)	Mesenchymal (1/6)	Neural (0/1)	Proneural (23/56)	Classical (4/9)	Mesenchymal (8/8)	Neural (2/3)	Proneural (5/5)	Classical (10/25)	Mesenchymal (13/41)	Neural (0/7)	Proneural (18/27)
	Precision	0.7	0.55	0	0.88	0.41	0.17	0	0.65	0.44	1	0.67	1	0.4	0.32	0	0.67
	Recall	0.82	0.71	0	0.74	0.86	0.04	0	0.74	1	0.73	0.4	1	0.36	0.54	0	0.58
	F1	0.76	0.62	0	0.8	0.13	0.06	0	0.69	0.61	0.84	0.5	1	0.38	0.4	0	0.62
	Avg F1	0.55				0.22				0.74				0.41			
	Accuracy	0.63				0.48				0.76				0.35			

iClusterPlus is a popular integration tool that has been used for multi-omics analysis of numerous cancers [20, 54, 55]. Given the poor performance of iClusterPlus in the binary classification of the HPC dataset, we speculated that the performance might improve if we restricted the methylation dataset to the top 1000 variable probes (variance range: 0.09–0.21) of the methylation dataset. As speculated, iClusterPlus showed improved classification accuracy in both the HPC and GBM datasets for similarly reduced datasets (Supplementary Table S8).

#### Performance in the presence of hidden classes

Independent analyses of multiple omics data types may identify subtypes that do not conform to one another as a subtype identified in one omics data may be undetectable in another omics data type. Such subtypes are referred to as the hidden classes, and the meta-dimensional tools aim to identify these classes through joint analysis of multi-omics data. The ability of the tools to retrieve a hidden class was tested using datasets A, B, C and D from the Small Group, where all datasets contain 60 samples and no noisy features. The dataset A that contained no hidden classes is simple compared to the other datasets, which are complex due to the presence of hidden classes in two or more omics data types.

**Figure 5:**
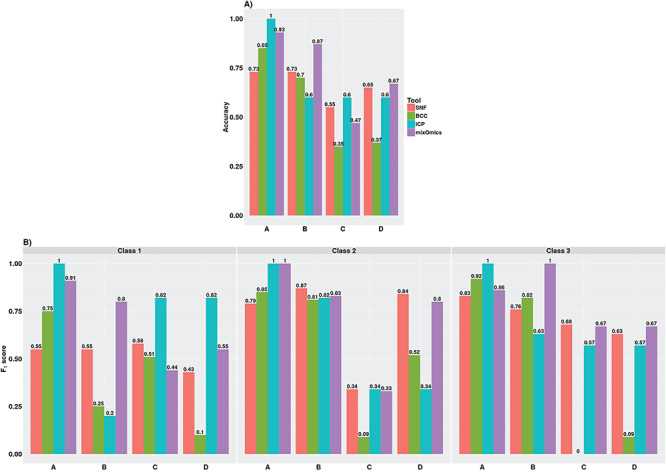
Performance results of meta-dimensional integration tools for datasets A–D in Small Group. (**A**) Accuracy measures and (**B**) F_1_ scores for individual classes. iCP – iClusterPlus.

The accuracies obtained by the tools for these datasets are shown in Figure 5A. Across all datasets, the performance of the tools was most accurate for dataset A. For the datasets A and C, the iClusterPlus tool obtained the highest accuracies (1 and 0.6, respectively), while for datasets B and D the mixOmics tool achieved the highest accuracies (0.87 and 0.67, respectively), as compared to the other tools.

The F_1_ scores achieved by these tools for individual classes in each dataset provide insights into the identification of the classes. A high F_1_ score, particularly for a hidden class, implies effective combining of shared and complementary subtype information from multiple omics data types to retrieve the underlying class. Our results showed that the recoverability of a hidden class by the tools was lower in comparison to a class that was not hidden. For example, the F_1_ scores achieved by the tools for Class 1 in datasets B and D were lower than that in datasets A and C (Figure 5B). The tools were more susceptible to missing the identification of a class when the class was simulated as a hidden class in more than one omics data type. In datasets C and D, Class 2 was simulated as a hidden class in two and one omics data types, respectively. All tools failed to identify Class 2 (F_1_ score < 0.5) in dataset C. However, in dataset D, the SNF and mixOmics tools were capable of identifying this class (F_1_ score > 0.5). For dataset D, which contained non-overlapping hidden classes in all three omics data types, only the mixOmics tool retrieved all three classes (F_1_ > 0.5), while the SNF and iClusterPlus tools retrieved two out of the three classes. The BCC tool performed the worst for complex datasets, such as datasets C and D, producing only a single cluster solution where all samples belonged to one class.

#### Accuracy of tools in small and large sample sizes

**Figure 6:**
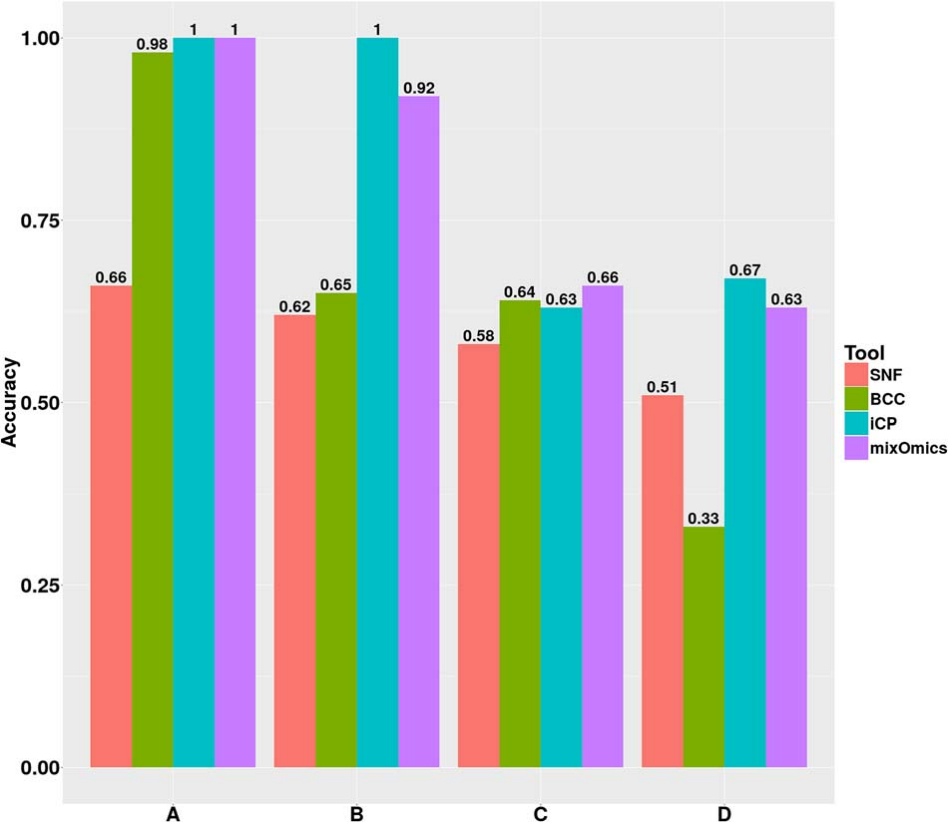
Accuracies achieved by the meta-dimensional integration tools for datasets A–D in Large Group. iCP – iClusterPlus.

The influence of sample size on the performance of the tools was assessed by comparing their performances using datasets from Small and Large Groups. Both groups contain the A, B, C and D datasets but differ only by sample size. The Small Group contains datasets with 60 samples whereas the Large Group contains datasets with 150 samples. The iClusterPlus and mixOmics tools accomplished more accurate classifications in the majority of datasets compared to SNF and BCC tools in the Large Group (Figure 6). When comparing the accuracies achieved by these tools for each dataset in Small and Large Groups, BCC, iClusterPlus and mixOmics tools showed improved classification accuracies for datasets in Large Group (Figures 5A and 6). Contrary to the other tools, the SNF tool displayed decreased classification performances for the majority of Large Group datasets. Lack of optimization of parameter settings for large sample sizes such as the number of neighbours while constructing the affinity graphs and number of iterations for fusing multiple networks may have contributed to the decreased performance of this tool.

#### Performance in the presence of noise

As noise is inherent in biological data, it is crucial to identify the robustness of the selected classifiers while processing noisy data. To test the robustness of these tools, we recreated the A, B, C and D datasets in Small and Large Groups with noisy features and referred to as Small Noisy Group and Large Noisy Group, respectively. Gaussian noisy features (mean = 0 and standard deviation = 2) were added to each of the omics data types in the datasets. As noisy features do not have profiles similar to that of the informative features, their presence confounds the classification process. A good classifier identifies the underlying classification despite the noisy features.

**Figure 7:**
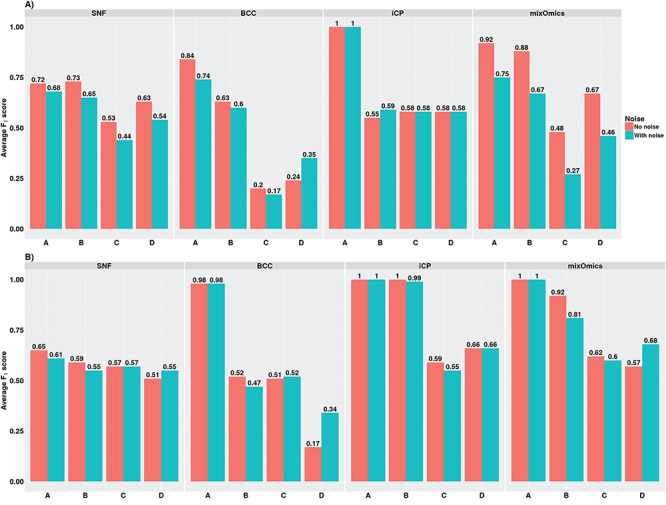
Performance results of meta-dimensional tools in datasets without noisy features versus datasets with noisy features. (**A**) Average F_1_ scores achieved for datasets in Small Group (no noise) and Small Noisy Group (with noise). (**B**) Average F_1_ scores achieved for datasets in Large Group (no noise) and Large Noisy Group (with noise). iCP – iClusterPlus.

First, we compared the accuracies achieved by the tools for the datasets in Small Group and Small Noisy Group (Figure 7A). The SNF, BCC and mixOmics tools showed reduced average F_1_ scores for datasets in the Small Noisy Group compared to that in the Small Group, demonstrating the lack of robustness to noise in these tools. The mixOmics tool was the most affected by the presence of noise compared to the other tools by showing the highest decrease of 43.75% for dataset C.

Next, we tested the influence of noise on these tools when noise was present in larger datasets by using the datasets in Large Group and Large Noisy Group. Contrary to the performance of the tools in small sample size datasets, they exhibited increased robustness as the sample size was increased. The decreases in the average F_1_ scores of all the tools for datasets in Large Noisy Group compared to that in Large Group were minimal or non-existent (Figure 7B). The presence of increased signal in these datasets may have contributed to the better performances. Overall, the iClusterPlus tool was the most resistant to noise, exhibiting no decrease in the performance in almost all the datasets with small and large sample sizes.

### Runtime of multi-staged and meta-dimensional integration tools

Given the large-scale sample availability and genome-wide sequencing of tens of thousands of genes, a trade-off between runtime and performance is highly desirable in a multi-omics integration tool. The runtime was recorded as the time taken to run the integration functions in each tool (Supplementary Tables S13 and S14). Among the multi-staged tools, Oncodrive-CIS took the longest time, followed by CNAmet. The iGC and MethylMix tools required less than a minute to complete the analysis. The PLRS, iGC and MethylMix tools were the more user-friendly of the multi-staged tools requiring minimal pre-processing and allowing for easy execution of functions. Among the meta-dimensional tools, iClusterPlus took the longest time, an average of 35 hours for the real datasets and 35 minutes for the simulated datasets. The SNF tool performed the meta-dimensional integration in less than 1 minute. The BCC and SNF tools were easy to execute as these tools had straight-forward functions requiring minimal steps. In contrast, running the iClusterPlus and mixOmics tools involved numerous steps, and user intervention was required to assess the results from each of the steps.

## Discussion

Our comparative study assessed the performance of five multi-staged and four meta-dimensional integration tools. These tools were designed to facilitate two common objectives for integrative analyses of cancer datasets: (i) identification of cancer driver genes and (ii) tumour subtype discovery or patient/sample classification.

In general, a multi-omics integration study encompasses the following steps: feature selection (optional), selection of an integration tool, processing of input data to fit the tool specifications, running the tool with optimized parameter settings, filtering the output (optional) and finally, interpretation of results. Identification of an optimal integration tool will minimize the challenges encountered at different steps and hence enable researchers to meet the integration objectives. Assessing the performance of the tools in identifying the cancer-associated genes, functions and pathways, and classification of tumour samples, are critical factors to be considered while choosing an efficient tool.

For multi-staged integration studies which seek to identify the cancer driving genes, our comparisons revealed that regression-based integration methods are most effective, identifying most cancer-associated genes, GO terms and pathways. For such analyses, we therefore recommend the PLRS and MethylMix tools as they implement regression-based integration and outperformed the other tools compared in this study. The regression-based integration approaches, which utilize continuous values for copy number or methylation status, prevent the loss of information due to the use of non-integer or sharp cut-off values. Using ordinal values can lead to reduced variability between the samples and hence miss the true relationship between the omics datasets analysed [56]. Cancer is a highly heterogeneous disease, and the use of ordinal values cannot accurately account for the differences in the degree of aberration among the samples and thus may not be optimal for integrative analyses [57].

As multi-staged integration tools focus on *cis* interactions between gene expression and copy number/methylation of genes and many cancer genes are implicated in the disease through multiple mechanisms, it is possible to miss the cancer genes in the tool results if small cut-offs, such as top 200 [58], are used. In order to not miss the identification of these cancer genes, we used a liberal cut-off that is larger than the number of the gold standard genes. While our assessment was restricted to the top 3000 most significant and informative genes, the performance of the tools was consistent when gene-level analysis was performed for top 1000 and top 2000 most significant genes (Supplementary Table S2). Users may also select genes based on the scores specific to each tool [15]. Our analyses also revealed potential for low precision of multi-staged integration tools [27]. Prior to the selection of the top 3000 significant genes from the output, in majority of the datasets, the PLRS predicted more than 8000 genes to have an association between copy number and gene expression (Supplementary Table S3). As multi-staged integration involves multiple testing, in order to reduce the false positives and prevent the loss of statistical power, we recommend either feature selection prior to integration or filtering down the output after integration.

To potentially improve the accuracy in identification of the true associations, we recommend the tools that utilize additional biological information during integration. The PLRS tool (the best performing method) uses both segmented copy number and thresholded copy number, as well as call probabilities when available. These data provide information on the heterogeneity among the samples as well as the precision of the copy number call, leading to the identification of statistically reliable associations that drive cancer [57]. Use of normal samples, when possible, may also add more reliability to the identified genes. For example, the use of normal samples allows the MethylMix tool to detect genes for which expression is not only regulated by methylation but also differentially methylated in comparison to normal samples [59]. A drawback common to all the multi-staged integration tools used in this study is that they model only the *cis* relationship between copy number or methylation and gene expression of genes and do not address the *trans* relationship, which is the regulation of distant genes, as has been reported in many studies [60, 61].

To classify the cancer subtypes using meta-dimensional integration, we recommend the mixOmics tool as it provided the most accurate classifications for the majority of the real and simulated datasets. Alternatively, when the sample labels are not known prior to integration, we recommend iClusterPlus as it provided most accurate classification for the majority of the simulated datasets. However, it should be noted that the performance of iClusterPlus tool can reduce in the presence of a large number of uninformative features (as seen for the real HPC and GBM datasets). Hence, feature selection prior to integration is critical. The iClusterPlus tool is also computationally intensive, and the runtime increases with an increase in the number of input features. A recent version of the iClusterPlus tool, iClusterBayes [62], endeavours to overcome these challenges using a Bayesian integrative clustering approach. The SNF tool may also be considered due to its enhanced performance in the real datasets, and its performance could potentially improve further by optimizing parameter settings [28]. However, the low accuracy of all the meta-dimensional tools in multi-class classification scenario (≤0.8) shows that improved integration approaches are needed.

An important characteristic of a good meta-dimensional integrative tool is to identify the subtypes that are common or unique to different omics datasets and effectively link this information to provide the best classification. Our study showed that the meta-dimensional tools have difficulties in identifying a class that is unidentifiable in the majority of the omics data types used for integration and classification. For the Tumour and PVTT classes in the HPC dataset and Neural class in GBM dataset, which are not clearly distinguished in the omics data types [43], all tools except mixOmics failed to retrieve these classes. Similarly, all tools showed low precision and recall scores for Class 2 in simulated dataset C in the Small Group, as this class was identifiable in only one of the three omics data types. When integrating such complex datasets, use of a tool that employs a supervised approach such as the mixOmics tool may provide better classification results as seen in the analysis with the real datasets and simulated dataset D. To estimate the complexity of the datasets and their influence on integration results, Tini *et al.* [28] recommends analysis and visualization of independent omics data types using techniques such as PCA to quantify the signal and subtypes present in them.

Our analyses showed that noise can have a substantial negative impact on the classification. Hence, use of advanced feature selection techniques is critical [63] prior to integration. Given, small sample sizes (~20 samples per class) and the presence of noise negatively impact the performance of these tools, use of larger sample sizes generally improves their performance due to the presence of higher amounts of signal relative to noise. However, due to the high dimensionality of these datasets, combined with heterogeneity in the samples, it is important to perform an initial power calculation to ensure sufficient sample sizes are analysed [64]. In datasets with a small number of noisy features, the iClusterPlus tool is preferable as the integrative results were least affected by noisy features.

## Limitations

All tools were run using default parameters to avoid bias. However, optimization of the parameters might have improved the performance of the tools, such as the SNF tool [28]. Only continuous data were used to assess the performance of meta-dimensional integrative tools. The iClusterPlus, mixOmics and SNF tools are also capable of integrating heterogeneous data, such as binary and categorical data types, and additional analyses are required to assess their performance when integrating such data. Our assessment does not fully reflect all the functional aspects of the tools. For instance, the CNAmet tool also includes quantification of the combined effect of copy number and methylation on gene expression, and the iClusterPlus and mixOmics tools can identify the features responsible for the classification; such functionalities were not assessed in the study. Cancer driver genes can be classified as protein-coding or non-coding. In our multi-staged analyses, due to the low number of non-coding genes in the input datasets (≤3%) and the gold standard cancer gene list (≤1%) (Supplementary Table S5), we could not accurately investigate the specific performance of the tools for non-coding genes.

## Conclusions

Our results provide much needed advice regarding selection and use of multi-omics integration tools. Tools implementing the regression-based approach for multi-staged integration effectively capture the association between the omics data types. The potential for false positive findings from multi-staged integration can be minimized using control datasets, feature selection prior to integration and/or filtering down the output after integration. For the meta-dimensional integration tools, the use of larger sample sizes enables more accurate identification of tumour and patient groups as well as diminishing the adverse effect of noise. Additional factors such as feature selection and parameter optimization are also critical and can improve the integration for both multi-staged and meta-dimensional integration tools. Although the multi-omics tools investigated improved the classification and feature identification that were undetectable in some individual omics data types, they still have much room for improvement with respect to their performance and ease of use.

## Supplementary Material

SupplementaryInformation_bbz121

## Notes

Key Points

- Multi-staged and meta-dimensional integration of multi-omics data achieve cancer driver gene identification and tumour subtyping, respectively.
- Regression-based approaches, such as the PLRS and MethylMix tools, provide more effective multi-staged integration due to their use of continuous values for copy number and methylation status.
- The mixOmics and iClusterPlus tools, which employ matrix factorization for meta-dimensional integration, showed superior classification compared to the other tools in real and simulated datasets.
- Prior feature selection and sufficient sample size are critical for effective multi-omics integration.

## Notes

**Anita Sathyanarayanan** is a PhD student in Bioinformatics at the Institute of Health and Biomedical Innovation and School of Biomedical Sciences, Queensland University of Technology, Australia.

## Notes

**Rohit Gupta** has a PhD in Computer Science with focus on data mining and machine learning applications in biology and medicine. His current research interests include Artificial Intelligence, Machine Learning, Bioinformatics, Genomics and Healthcare Informatics.

## Notes

**Erik W. Thompson** is a professor in Breast Cancer Research in the School of Biomedical Sciences at Queensland University of Technology, Australia, and the Associate Director of the Institute of Health and Biomedical Innovation, Queensland University of Technology, at the Translational Research Institute, Australia.

## Notes

**Dale R. Nyholt** is a professor in the School of Biomedical Sciences, Faculty of Health, and Institute of Health and Biomedical Innovation at the Queensland University of Technology, Brisbane, Australia.

## Notes

**Denis Bauer** is a group leader at CSIRO, with background in genomics and machine learning. Her current research interests include medical genomics, Apache Spark-based genome-wide variant association, genome engineering and serverless cloud architecture for life science.

## Notes

**Shivashankar H. Nagaraj** is an Advance Queensland research fellow at the Institute of Health and Biomedical Innovation and School of Biomedical Sciences, Queensland University of Technology, Australia and is also at the Translational Research Institute, Australia.
