# Supplementary material for: A comparative study of multi-omics integration tools for cancer driver gene identification and tumour subtyping
Source: Brief Bioinform. 2019 Nov 27;21(6):1920–36. doi: 10.1093/bib/bbz121 (PMC7711266; doi:10.1093/bib/bbz121)
Supplement: SupplementaryInformation_bbz121 [file supplementaryinformation_bbz121.docx]

# Supplementary information

**Summary of multi-staged integration tools**

CNAmet

This tool models the effect of copy number and methylation aberration on gene expression. The primary focus is to identify genes which have upregulated expression due to amplification and hypomethylation or downregulated expression due to deletion and hypermethylation. However, independent associations between copy number aberration and gene expression and methylation change and gene expression can also be modelled. To identify such genes, the algorithm calculates three estimates: (i) Weights: Two weight measures are calculated, for copy number and methylation variation. The weights inform the ratio of signal to noise and are based on the mean and standard deviation of gene expression in normal samples and samples with variation (ii) Score: The two weights are summed to get the score. Correction of the score can be performed by multiplying with the ratio of samples with both amplification and hypomethylation (or deletion and hypermethylation) to total number of samples. (iii) Significance value: The p-value of the score is calculated using the permutation test and adjusted using the FDR method.

The method is available as an R package and as a component in Anduril framework. As the method only utilises only binary information on the gene copy number aberration and methylation status, information may be lost due to lack of association between the degree of aberration and gene expression.

iGC

This tool uses the Student’s t-test to identify the genes with significant differential expression due to copy number alterations. For each gene based on its copy number status, the samples are classified as gain, loss, and neutral. If the fraction of gain or loss samples is greater than a defined cut-off (default is 20%), the gene is labelled as gain or loss, respectively. Lastly, the expression levels of gain or loss genes are compared to that in the rest of the samples in the dataset using the Student’s t-test with unequal variance. The method is implemented as an R/Bioconductor package.

PLRS

PLRS or Piecewise Linear Regression Splines is another tool that models the *cis* relationship between copy number and expression of a gene using piecewise linear regression splines [1]. It reports genes that reject the null hypothesis that copy number does not affect gene expression. Multiple linear associations for different copy number states address the variations in the relationship between gene expressions and copy numbers for each gene. Unlike other methods, PLRS utilises the copy number information, thresholded copy number, as well as call probabilities if available to establish the relationship. The tool carries out model fitting, model selection, and provides confidence intervals. The models can be saved and visually inspected for identifying relevant genes. The tool is available in R/Bioconductor. It can also be employed to model the relationship between miRNA and copy number.

Oncodrive-CIS

This statistical framework performs gene-centric integration of copy number and expression data to detect the likely driver genes based on the rationale that driver genes are more likely to cause a larger *cis* change in expression than the passenger genes [2]. The framework first estimates the impact of copy number change on gene expression for each gene with respect to normal samples and tumour samples with diploid tumours. This is followed by estimation of two standard scores that measure (i) bias towards expression dysregulation in tumour samples with copy number aberrations in comparison to diploid tumours, (ii) bias towards expression dysregulation in tumour samples with copy number aberrations in comparison to normal samples. The scores are combined followed by ranking of genes based on the combined score. Genes with higher ranks have a larger bias towards dysregulation of gene expression due to change in copy number and are the likely driver genes. The tool is available as a python script and has to be executed via the terminal (command line).

MethylMix

This tool focuses on modelling the regulatory influence of methylation on gene expression. The algorithm can be broadly divided into three steps: (i) A linear regression model is built for gene expression using the beta methylation values of the CpG site (or a cluster of correlated sites) associated with the gene. If they are inversely correlated and the gene expression can be predicted using the regression model, then such genes, called as the transcriptionally predictive genes, are selected. (ii) Different methylation states of the genes are identified by fitting univariate beta mixture models. (iii) A novel metric called as ‘Differential Methylation value’ is estimated as the difference between the mean methylation level of each state in diseased samples and normal samples. The output is a set of transcriptionally predictive and differentially methylated genes. Application of this tool in pan-cancer has been shown [3] and can be applied to other diseases as well. It is available as an R/Bioconductor package.

**Summary of multi-staged integration tools**

SNF (Similarity Network Fusion)

This unsupervised integration method integrates various data types ranging from omics data to clinical meta-data, image data, etc. The algorithm is network-based and is an example of transformation-based integration tools. It involves two broad steps: (i) For each molecular data, a patient status matrix indicating similarity of each patient to all others is calculated. Additionally, a kernel matrix, based on K nearest neighbours method, containing the similarity of the patients with other patients belonging to the same neighbourhood is created. (ii) Subtyping based on fused matrix: Given the status matrix and kernel matrix for each data type, each status matrix is iteratively updated using its own kernel matrix and the status matrices from other data types. Finally the overall status matrix is obtained by taking an average over all the status matrices and the cluster assignment is done using spectral clustering. Although this method can integrate various data types, it is not designed to identify the important features contributing to the clustering. The tool is available for both R and Matlab.

Bayesian Consensus Clustering

Bayesian Consensus Clustering (BCC), based on a Bayesian integrative approach, identifies clustering of independent data types as well as an overall integrated clustering by capturing common and unique patterns. The underlying assumption is that clusters of independent data types loosely adhere to the overall integrated clusters. It is implemented by extending the Dirichlet mixture model. Gibbs sampling is performed to determine the data type-specific clusters and integrated clusters. The algorithm does not assume any prior distribution for the data types and hence allows uncertainty in all parameters. In comparison to SNF, the data type specific clustering and the consensus clustering are estimated simultaneously. The strength of association between the individual data types and the integrated clustering is estimated and accounted for in the model. The method is available as an R package.

iClusterPlus

It is an enhanced version of iCluster [4]. In contrast to iCluster, iClusterPlus can handle different data types. The method can simultaneously perform integration, clustering, and feature selection. It is based on joint latent variable clustering where the cancer driving factors are represented as latent variables. The omic variables and the latent variables are connected by parametric modelling based on the omic data type. For example, mutation information represented as binary data is modelled using logistic regression, copy number data (categorical data type) using multilogit regression, and gene expression (continuous data type) using linear regression. Feature selection is performed using Lasso penalised log likelihood estimation. Finally K-means clustering is performed to identify the underlying subtypes. Both iCluster and iClusterPlus have been used in several key cancer analyses to infer the subtypes from the data [5-7]. The method is available as an R/Bioconductor package.

mixOmics

It is a holistic package for various kinds of analysis and data integration. The DIABLO framework [8] is used for integration of multiple omics across several samples. It implements a generalisation of partial least square for supervised analysis. It involves identification of the optimal number of components and number of gene features from each omics in each component to create a prediction model. The results include extensive graphical outputs, such as a diagnostic plot showing the correlation between the data in each component, a plot to visualise the samples in the space spanned by each block, and several other plots to visualise the relationship between the selected variables. The method is available as an R package.

**Multi-staged integration tools analyses**

**Integration of copy number and gene expression**

**Datasets used**

Table S1: Details on the number of features in each omics data in the datasets used for multi-staged analysis

| **Dataset** | **Omics data** | **# features** | **# samples** | **Download URL** |
| --- | --- | --- | --- | --- |
| Mesothelioma dataset | Gene expression | 20530 | 87 | https://tcga.xenahubs.net/download/TCGA.MESO.sampleMap/HiSeqV2.gz |
|  | Copy number variation (Thresholded) | 24776 | 87 | https://tcga.xenahubs.net/download/TCGA.MESO.sampleMap/Gistic2_CopyNumber_Gistic2_all_thresholded.by_genes.gz |
|  | Copy number variation (Segmented) | 24776 | 87 | https://tcga.xenahubs.net/download/TCGA.MESO.sampleMap/Gistic2_CopyNumber_Gistic2_all_data_by_genes.gz |
|  | Methylation | 20117 | 87 | http://gdac.broadinstitute.org/runs/stddata__2016_01_28/data/MESO/20160128/gdac.broadinstitute.org_MESO.Methylation_Preprocess.Level_3.2016012800.0.0.tar.gz |
| Pancreatic cancer dataset | Gene expression | 20530 | 183 | https://tcga.xenahubs.net/download/TCGA.PAAD.sampleMap/HiSeqV2.gz |
|  | Copy number variation (Thresholded) | 24776 | 184 | https://tcga.xenahubs.net/download/TCGA.PAAD.sampleMap/Gistic2_CopyNumber_Gistic2_all_thresholded.by_genes.gz |
|  | Copy number variation (Segmented) | 24776 | 184 | https://tcga.xenahubs.net/download/TCGA.PAAD.sampleMap/Gistic2_CopyNumber_Gistic2_all_data_by_genes.gz |
|  | Methylation | 20089 | 195 | http://gdac.broadinstitute.org/runs/stddata__2016_01_28/data/PAAD/20160128/gdac.broadinstitute.org_PAAD.Methylation_Preprocess.Level_3.2016012800.0.0.tar.gz |
| Colon cancer dataset | Gene expression | 20530 | 329 | https://tcga.xenahubs.net/download/TCGA.COAD.sampleMap/HiSeqV2.gz |
|  | Copy number variation (Thresholded) | 24776 | 329 | https://tcga.xenahubs.net/download/TCGA.COAD.sampleMap/Gistic2_CopyNumber_Gistic2_all_thresholded.by_genes.gz |
|  | Copy number variation (Segmented) | 24776 | 451 | https://tcga.xenahubs.net/download/TCGA.COAD.sampleMap/Gistic2_CopyNumber_Gistic2_all_data_by_genes.gz |
|  | Methylation | 20116 | 335 | http://gdac.broadinstitute.org/runs/stddata__2016_01_28/data/COAD/20160128/gdac.broadinstitute.org_COAD.Methylation_Preprocess.Level_3.2016012800.0.0.tar.gz |
| Melanoma dataset | Gene expression | 20530 | 474 | https://tcga.xenahubs.net/download/TCGA.SKCM.sampleMap/HiSeqV2.gz |
|  | Copy number variation (Thresholded) | 24776 | 367 | https://tcga.xenahubs.net/download/TCGA.SKCM.sampleMap/Gistic2_CopyNumber_Gistic2_all_thresholded.by_genes.gz |
|  | Copy number variation (Segmented) | 24776 | 367 | https://tcga.xenahubs.net/download/TCGA.SKCM.sampleMap/Gistic2_CopyNumber_Gistic2_all_data_by_genes.gz |
|  | Methylation | 20110 | 475 | http://gdac.broadinstitute.org/runs/stddata__2016_01_28/data/SKCM/20160128/gdac.broadinstitute.org_SKCM.Methylation_Preprocess.Level_3.2016012800.0.0.tar.gz |

**Results of copy number and gene expression integration**

Table S2: The number of overlapping CCGL genes in the top 1000, top 2000, top 3000, and all significant gene results identified by the tools in the four cancer datasets. Top 1000 significant genes include top 500 amplification and top 500 deletion genes; top 2000 significant genes include top 1000 amplification and top 1000 deletion genes; top 3000 genes include top 1500 amplification and 1500 deletion genes. The numbers in the parantheses in the All significant genes column indicate the % of overlapping CCGL genes present in all the significant genes identified by the tools.

| Tool | Top 1000 significant genes | Top 2000 significant genes | Top 3000 significant genes | All significant genes |
| --- | --- | --- | --- | --- |
|  | Mesothelioma dataset | | | |
| CNAmet | 47 | 91 | 103 | 114 (5.02) |
| iGC | 40 | 63 | 63 | 63 (5.45) |
| PLRS | **53** | **101** | **124** | 124 (5.02) |
| Oncodrive-CIS | 42 | 43 | 43 | 43 (6.49) |
|  | Pancreatic cancer dataset | | | |
| CNAmet | 57 | 101 | 147 | 440 (3.80) |
| iGC | 56 | **114** | 138 | 199 (2.30) |
| PLRS | **60** | 107 | **180** | 471 (3.98) |
| Oncodrive-CIS | 53 | 108 | 122 | 122 (2.42) |
|  | Colon cancer dataset | | | |
| CNAmet | 46 | 90 | 146 | 462 (5.71) |
| iGC | 58 | 115 | 181 | 305 (5.77) |
| PLRS | 62 | **125** | **188** | 449 (5.59) |
| Oncodrive-CIS | **65** | 108 | 165 | 177 (5.99) |
|  | Melanoma dataset | | | |
| CNAmet | 47 | 101 | 140 | 668 (8.37) |
| iGC | 61 | 124 | 172 | 508 (14.65) |
| PLRS | **82** | **137** | **198** | 697 (8.33) |
| Oncodrive-CIS | 68 | 126 | 181 | 318 (14.79) |

Table S3: Results from the multi-staged integration tools integration copy number and gene expression prior to selection of top 1500 amplification and deletion driven genes

| Tool | Input # genes | Input # samples | Amplification genes (FDR <0.05) | Deletion genes (FDR <0.05) | Both (FDR <0.05) | Total genes with FDR <0.05 | % of input genes |
| --- | --- | --- | --- | --- | --- | --- | --- |
| Mesothelioma dataset | | | | | | | |
| CNAmet | 8372 | 87 | 457 | 1553 | 262 | 2272 | 27.14 |
| iGC | 8372 | 87 | 231 | 924 | 0 | 1155 | 13.80 |
| PLRS | 8372 | 87 | NA | NA | NA | 2472 | 29.53 |
| Oncodrive-CIS | 8372 | 87 | 131 | 519 | 13 | 663 | 7.92 |
| Pancreatic cancer dataset | | | | | | | |
| CNAmet | 12977 | 177 | 1139 | 1745 | 8690 | 11574 | 89.19 |
| iGC | 12977 | 177 | 3354 | 4783 | 534 | 8671 | 66.82 |
| PLRS | 12977 | 177 | NA | NA | NA | 11849 | 91.30 |
| Oncodrive-CIS | 12977 | 177 | 1343 | 2491 | 1200 | 5034 | 38.80 |
| Colon cancer dataset | | | | | | | |
| CNAmet | 12358 | 276 | 2266 | 1613 | 4205 | 8084 | 65.42 |
| iGC | 12358 | 276 | 2877 | 2241 | 167 | 5285 | 42.77 |
| PLRS | 12358 | 276 | NA | NA | NA | 8039 | 65.05 |
| Oncodrive-CIS | 12358 | 276 | 1186 | 1439 | 332 | 2957 | 23.93 |
| Melanoma dataset | | | | | | | |
| CNAmet | 14745 | 366 | 1111 | 4058 | 2816 | 7985 | 53.54 |
| iGC | 14745 | 366 | 992 | 2468 | 8 | 3468 | 23.52 |
| PLRS | 14745 | 366 | NA | NA | NA | 8369 | 56.76 |
| Oncodrive-CIS | 14745 | 366 | 1102 | 871 | 117 | 2150 | 14.58 |

**Venn intersection results for copy number and gene expression integration tools for mesothelioma, pancreatic cancer, colon cancer and melanoma datasets**

**Biological Process (BP) gene ontology (GO) terms**


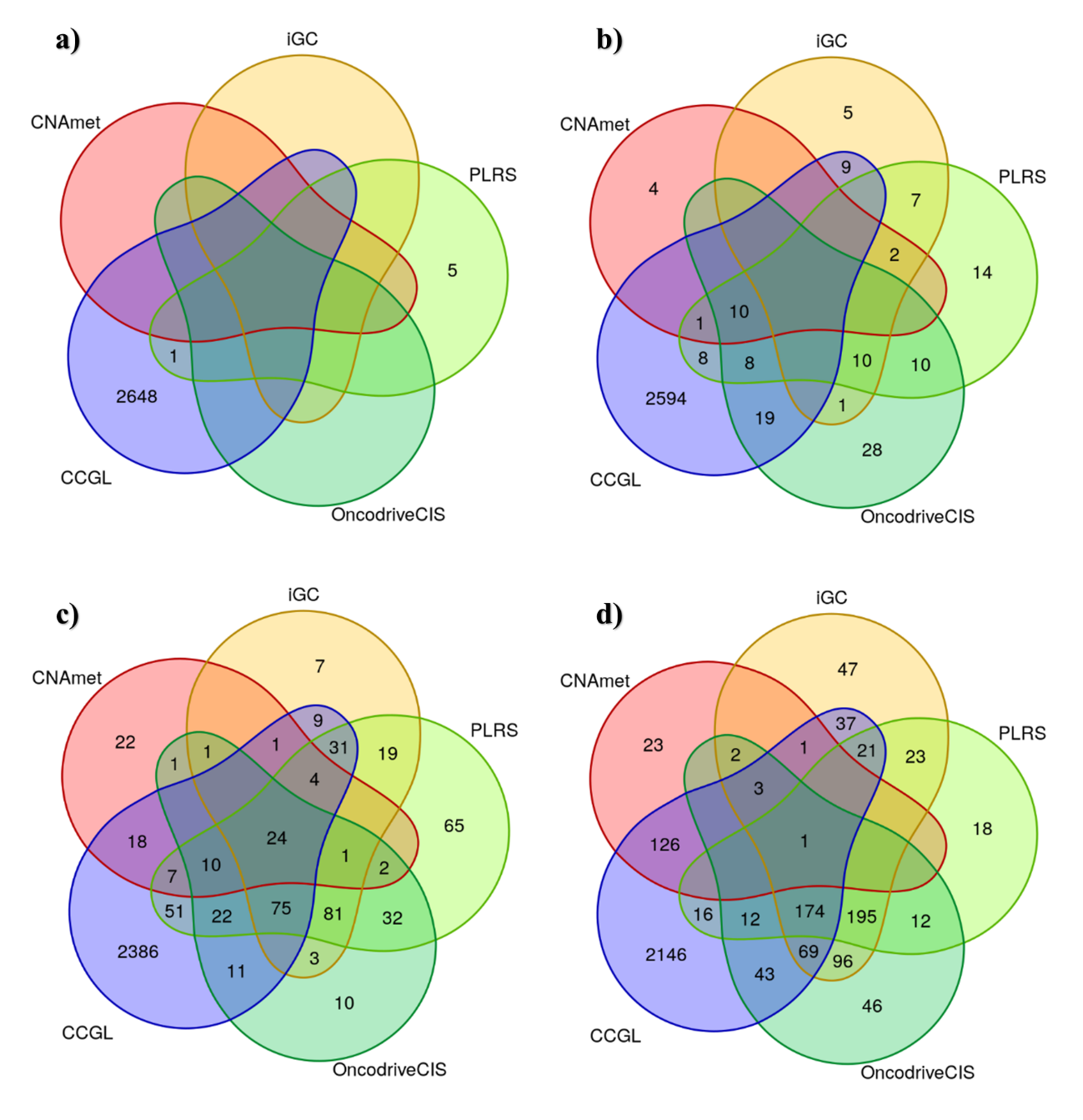


Figure S1: Congruence between the tools and GGCL for GO BP terms in a) mesothelioma dataset b) pancreatic cancer dataset c) colon cancer dataset d) melanoma dataset

**Cellular Component (CC) gene ontology (GO) terms**


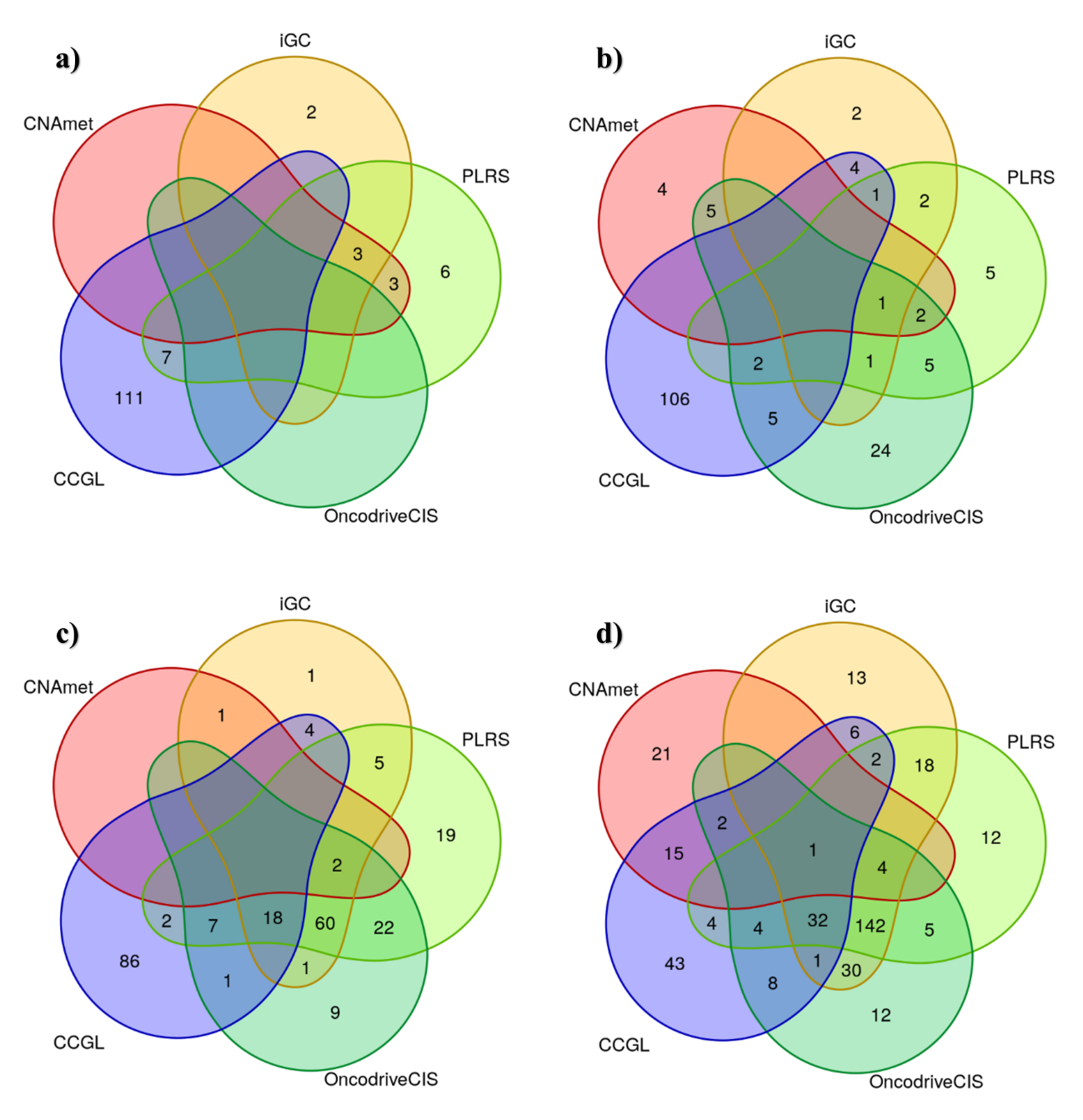


Figure S2: Congruence between the tools and GGCL for GO CC terms in a) mesothelioma dataset b) pancreatic cancer dataset c) colon cancer dataset d) melanoma dataset

**Molecular Functions (MF) gene ontology (GO) terms**


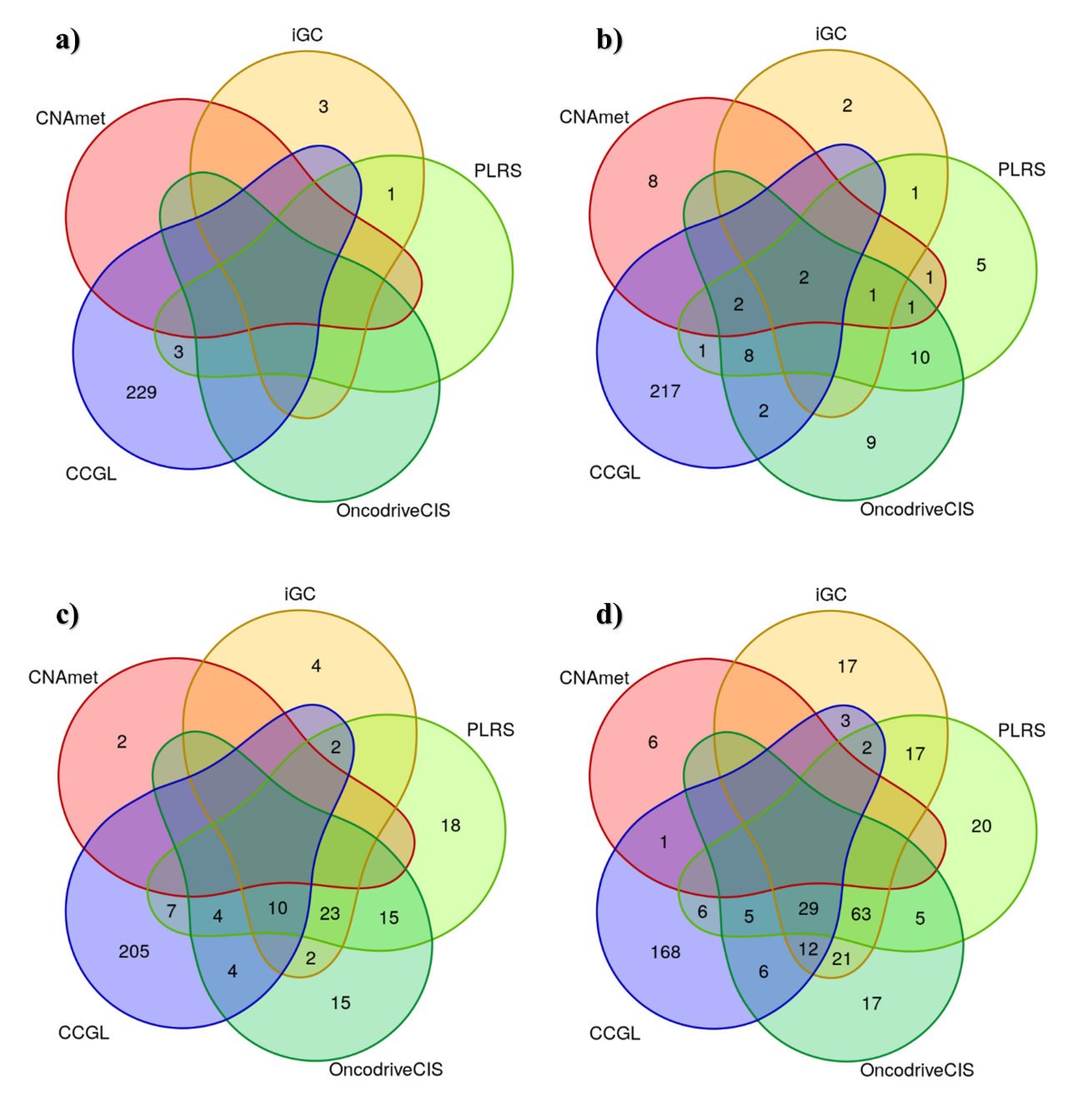


Figure S3: Congruence between the tools and GGCL for GO MF terms in a) mesothelioma dataset b) pancreatic cancer dataset c) colon cancer dataset d) melanoma dataset

**Reactome Pathway terms**


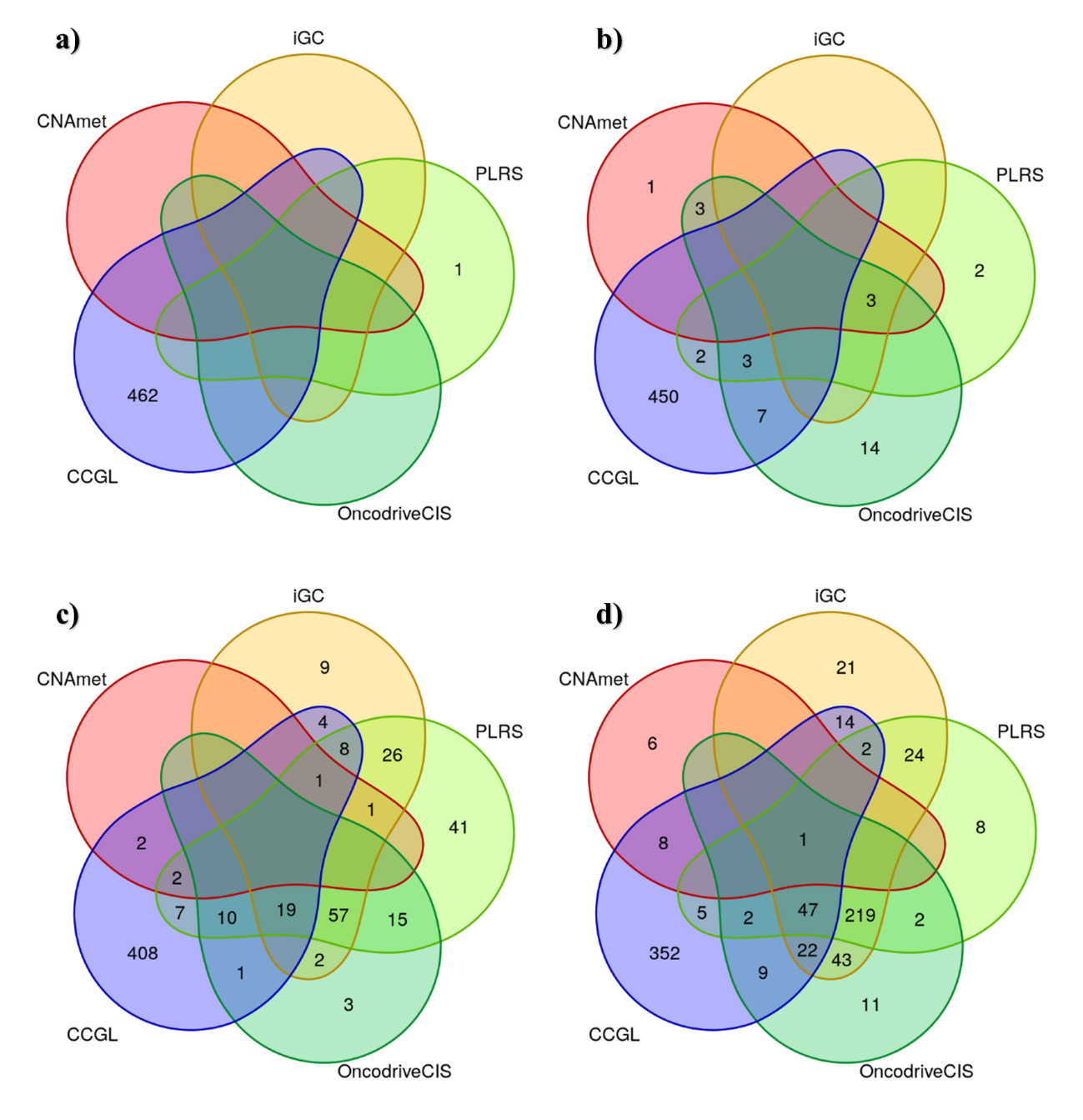


Figure S4: Congruence between the tools and GGCL for Reactome pathways in a) mesothelioma dataset b) pancreatic cancer dataset c) colon cancer dataset d) melanoma dataset

**Results of methylation and gene expression integration**

Table S4: Results from the multi-staged integration tools integration methylation and gene expression prior to selection of top 1500 hypomethylation and hypermethylation driven genes

| Tool | Input # genes | Input # samples | Hypomethylation (FDR < 0.05) | Hypermethylation (FDR <0.05) | Both (FDR <0.05) | Total | % of input genes |
| --- | --- | --- | --- | --- | --- | --- | --- |
| Mesothelioma dataset | | | | | | | |
| CNAmet | 16114 | 87 | 588 | 367 | 1 | 956 | 5.93 |
| MethylMix | 16114 | 87 | NA | NA | NA | 2346 | 14.56 |
| Pancreatic cancer dataset | | | | | | | |
| CNAmet | 16144 | 177 | 1183 | 575 | 13 | 1771 | 10.97 |
| MethylMix | 16144 | 177 | NA | NA | NA | 3025 | 18.74 |
| Colon cancer dataset | | | | | | | |
| CNAmet | 15935 | 276 | 1347 | 512 | 27 | 1886 | 11.84 |
| MethylMix | 15935 | 276 | NA | NA | NA | 2156 | 13.53 |
| Melanoma dataset | | | | | | | |
| CNAmet | 16045 | 366 | 1790 | 1657 | 247 | 3694 | 23.02 |
| MethylMix | 16045 | 366 | NA | NA | NA | 2492 | 15.53 |

**Venn intersection results for methylation and gene expression integration tools in mesothelioma, pancreatic cancer, colon cancer and melanoma datasets**


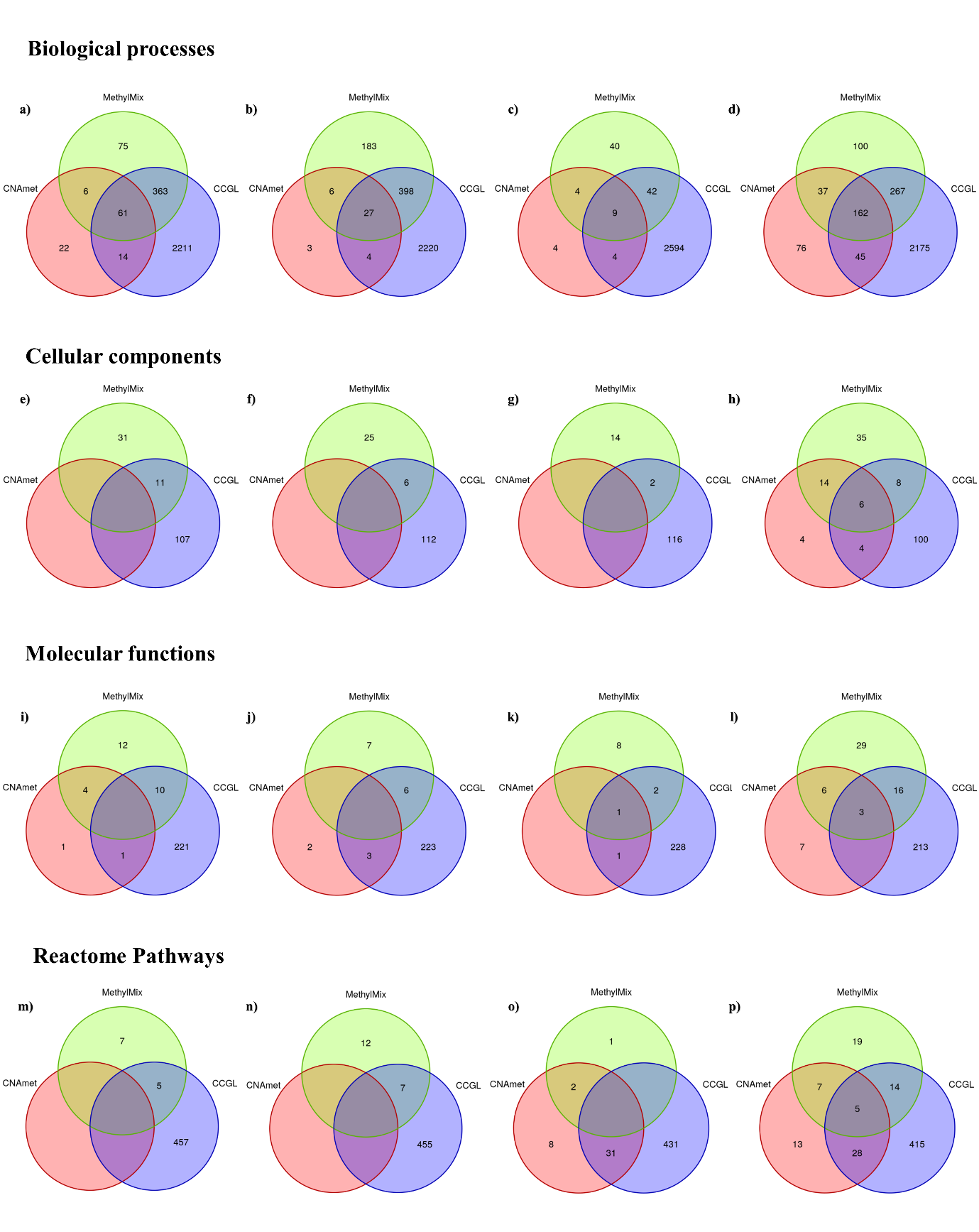


Figure S5: Congruence between CNAmet and MethylMix tools and CCGL for mesothelioma, pancreatic cancer, colon cancer and melanoma datasets for GO BP terms a – d; for GO CC terms e – h; GO MF terms i – l; pathways m – p. No numbers indicate that no entities were present or were found in common.

**Non-coding genes in colon cancer dataset and tools results**

Table S5: Genetic classification of the genes in the input dataset, CCGL list, and output for tools integrating copy number and gene expression. The genetic classification was downloaded from HNGC Mart (<https://biomart.genenames.org/martform/#!/default/HGNC?datasets=hgnc_gene_mart>)

|  | Input genes | Cancer genes | CNAmet | iGC | PLRS | OncodriveCIS |
| --- | --- | --- | --- | --- | --- | --- |
| Protein Coding | 12005 | 960 | 144 | 178 | 185 | 163 |
| Noncoding | 112 | 6 | 2 | 1 | 1 | 1 |
| Phenotype only | 0 | 0 | 0 | 0 | 0 | 0 |
| Pseudogene | 121 | 3 | 0 | 0 | 0 | 0 |
| Other | 87 | 2 | 0 | 0 | 0 | 0 |
| Ambiguous | 32 | 10 | 0 | 2 | 2 | 1 |

Ambiguous genes are those genes with names that overlap among other classes

**Meta-dimensional integration tools analyses**

**Datasets used**

Table S6: Details on the number of features, samples and inter-sample variance feature selection in each omics data in the real datasets used for multi-staged analysis

| **Dataset** | **Omics data** | **# of features** | **# of samples** | **Feature selection variance cut-off (%)** | **# features after selection** | **Download URL** |
| --- | --- | --- | --- | --- | --- | --- |
| Hepatocellular carcinoma dataset | Gene expression | 22827 | 60 | 10 | 1728 | https://www.ncbi.nlm.nih.gov/geo/query/acc.cgi?acc=GSE77509 |
|  | miRNA expression | 2578 | 60 | 10 | 60 | https://www.ncbi.nlm.nih.gov/geo/query/acc.cgi?acc=GSE76903 |
|  | Methylation | 485577 | 60 | 5 | 24272 | https://www.ncbi.nlm.nih.gov/geo/query/acc.cgi?acc=GSE77269 |
| Glioblastoma dataset | Gene expression | 12042 | 539 | 25 | 3011 | https://tcga.xenahubs.net/download/TCGA.GBM.sampleMap/HT_HG-U133A.gz |
|  | Methylation | 27578 | 288 | 25 | 5744 | https://tcga.xenahubs.net/download/TCGA.GBM.sampleMap/HumanMethylation27.gz |
|  | Protein expression | 131 | 215 | 25 | 33 | https://tcga.xenahubs.net/download/TCGA.GBM.sampleMap/RPPA_RBN.gz |

**Feature selection in hepatocellular carcinoma and glioblastoma datasets using inter-sample variance**

**Hepatocellular carcinoma dataset**


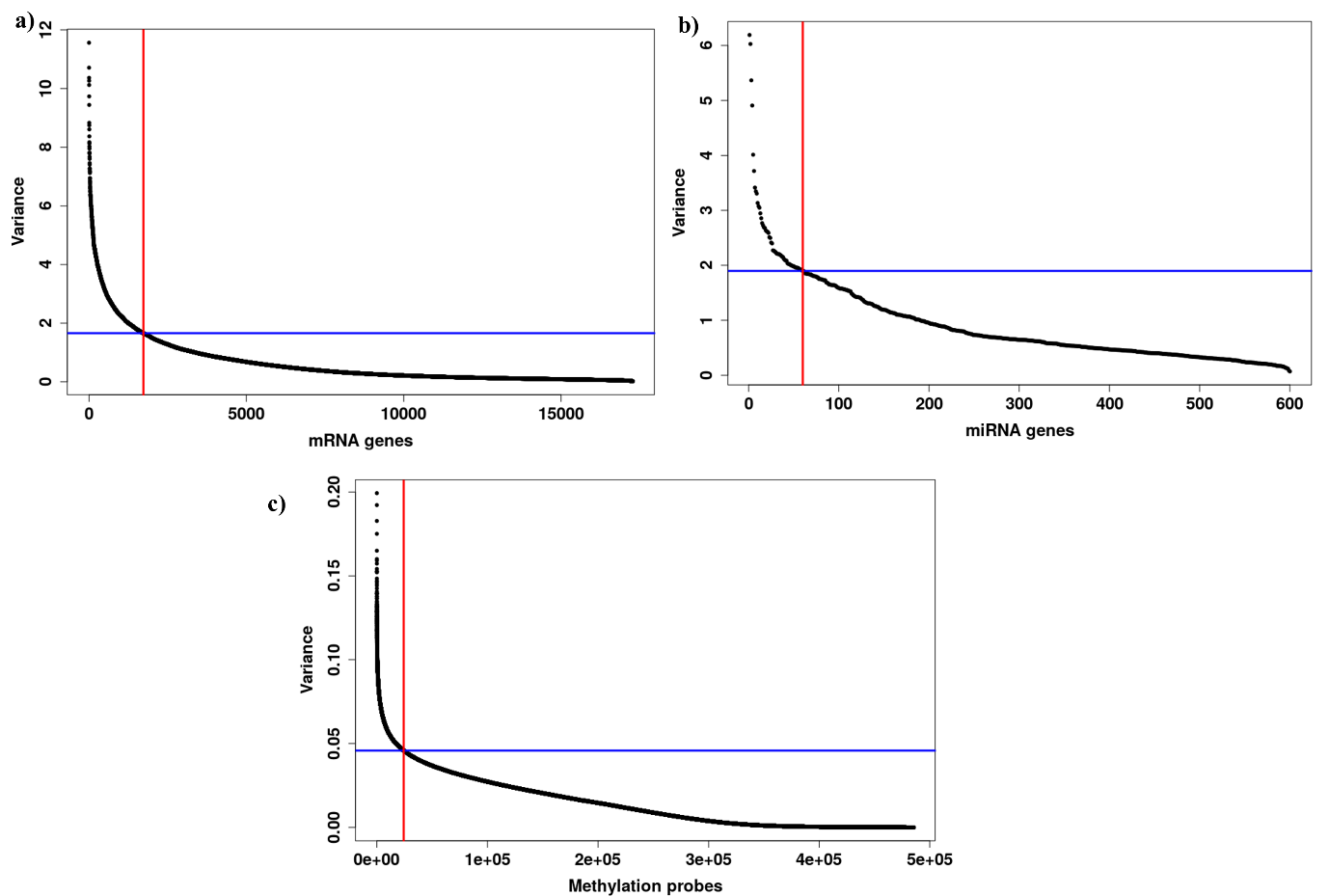


Figure S6: Feature selection of hepatocellular carcinoma dataset based on inter-sample variance. The features to the left of red line and top of the blue line were selected for meta-dimensional integration. a) Top 10% variable mRNA genes; b) Top 10% variable miRNA genes; c) Top 5% variable methylation probes

**Glioblastoma dataset**


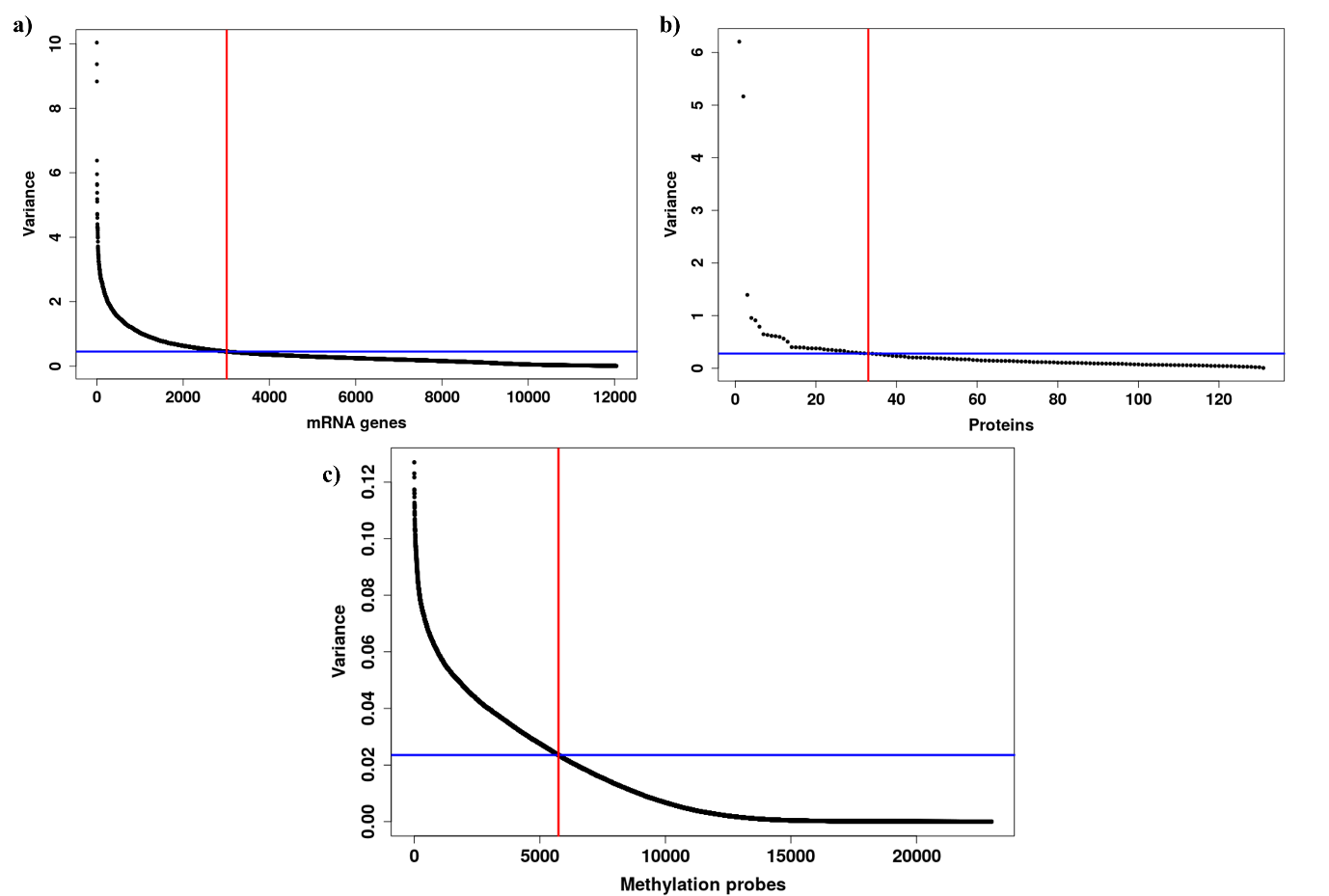


Figure S7: Feature selection of glioblastoma dataset based on inter-sample variance. The features to the left of red line and top of the blue line were selected for meta-dimensional integration. a) Top 25% variable mRNA genes; b) Top 25% variable proteins; c) Top 25% variable methylation probes

**TCGA breast cancer dataset used for simulated datasets**

Table S7: Number of features in each omics breast cancer dataset downloaded from TCGA

| **Omics data in TCGA BRCA dataset** | **Number of features** | **Download URL** |
| --- | --- | --- |
| Gene expression | 17814 | http://tcga-data.nci.nih.gov/docs/publications/brca_2012/BRCA.exp.348.med.txt |
| miRNA | 1222 | http://tcga-data.nci.nih.gov/docs/publications/brca_2012/BRCA.348.mimat.txt |
| Methylation | 574 | http://tcga-data.nci.nih.gov/docs/publications/brca_2012/BRCA.Methylation.574probes.802.txt |

**iClusterPlus Results**

Table S8: iClusterPlus results with reduced input features

| Dataset | Input data | | Metrics/Tools | iClusterPlus | | | |
| --- | --- | --- | --- | --- | --- | --- | --- |
| HPC – Binary Classification | Omics | Samples x Features | Classes | Normal (20/22) |  | PVTT/Tumour (38/38) |  |
|  | mRNA | 60 x 1728 | Precision | 0.91 |  | 1 |  |
|  | miRNA | 60 x 60 | Recall | 1 |  | 0.95 |  |
|  | Methylation | 60 x 1000 | F1 | 0.95 |  | 0.97 |  |
|  |  |  | Avg F1 | 0.96 | | | |
|  |  |  | Accuracy | 0.97 | | | |
| HPC – Multi-class classification | Omics | Samples x Features | Classes | Normal (20/22) | PVTT (10/19) | Tumour (10/19) |  |
|  | mRNA | 60 x 1728 | Precision | 0.91 | 0.53 | 0.53 |  |
|  | miRNA | 60 x 60 | Recall | 1 | 0.5 | 0.5 |  |
|  | Methylation | 60 x 1000 | F1 | 0.95 | 0.51 | 0.51 |  |
|  |  |  | Avg F1 | 0.66 | | | |
|  |  |  | Accuracy | 0.66 | | | |
| GBM – Multi-class classification | Omics | Samples x Features | Classes | Classsical (13/29) | Mesenchymal (10/28) | Neural (7/32) | Proneural (11/11) |
|  | mRNA | 100 x 1000 | Precision | 0.45 | 0.36 | 0.22 | 1 |
|  | Protein | 100 x 131 | Recall | 0.46 | 0.42 | 0.41 | 0.35 |
|  | Methylation | 100 x 1000 | F1 | 0.45 | 0.39 | 0.29 | 0.52 |
|  |  |  | Avg F1 | 0.41 | | | |
|  |  |  | Accuracy | 0.68 | | | |

**Meta-dimensional integration tools classification results for tools on simulated datasets**

Table S9: Precision, Recall, F_1_ scores for individual classes along with average F_1_ and accuracy scores for Small datasets group

| Tool | Dataset | Class 1 | | | Class 2 | | | Class 3 | | | Average F_1_ | Accuracy |
| --- | --- | --- | --- | --- | --- | --- | --- | --- | --- | --- | --- | --- |
|  |  | Precision | Recall | F_1_ | Precision | Recall | F_1_ | Precision | Recall | F_1_ |  |  |
| SNF | A | 0.62 | 0.5 | 0.55 | 0.74 | 0.85 | 0.79 | 0.81 | 0.85 | 0.83 | 0.72 | 0.73 |
| SNF | B | 0.62 | 0.5 | 0.55 | 0.89 | 0.85 | 0.87 | 0.68 | 0.85 | 0.76 | 0.73 | 0.73 |
| SNF | C | 0.61 | 0.55 | 0.58 | 0.4 | 0.3 | 0.34 | 0.59 | 0.8 | 0.68 | 0.53 | 0.55 |
| SNF | D | 0.47 | 0.4 | 0.43 | 0.76 | 0.95 | 0.84 | 0.67 | 0.6 | 0.63 | 0.63 | 0.65 |
| BCC | A | 1 | 0.6 | 0.75 | 0.74 | 1 | 0.85 | 0.9 | 0.95 | 0.92 | 0.84 | 0.85 |
| BCC | B | 0.75 | 0.15 | 0.25 | 0.7 | 0.95 | 0.81 | 0.69 | 1 | 0.82 | 0.63 | 0.7 |
| BCC | C | 0.34 | 1 | 0.51 | 0.5 | 0.05 | 0.09 | 0 | 0 | 0 | 0.2 | 0.35 |
| BCC | D | 1 | 0.05 | 0.1 | 0.35 | 1 | 0.52 | 0.5 | 0.05 | 0.09 | 0.24 | 0.37 |
| iClusterPlus | A | 1 | 1 | 1 | 1 | 1 | 1 | 1 | 1 | 1 | 1 | 1 |
| iClusterPlus | B | 0.3 | 0.15 | 0.2 | 0.69 | 1 | 0.82 | 0.62 | 0.65 | 0.63 | 0.55 | 0.6 |
| iClusterPlus | C | 0.69 | 1 | 0.82 | 0.38 | 0.3 | 0.34 | 0.67 | 0.5 | 0.57 | 0.58 | 0.6 |
| iClusterPlus | D | 0.69 | 1 | 0.82 | 0.38 | 0.3 | 0.34 | 0.67 | 0.5 | 0.57 | 0.58 | 0.6 |
| mixOmics | A | 0.83 | 1 | 0.91 | 1 | 1 | 1 | 1 | 0.75 | 0.86 | 0.92 | 0.93 |
| mixOmics | B | 0.8 | 0.8 | 0.8 | 0.83 | 0.83 | 0.83 | 1 | 1 | 1 | 0.88 | 0.87 |
| mixOmics | C | 0.5 | 0.4 | 0.44 | 0.33 | 0.33 | 0.33 | 0.6 | 0.75 | 0.67 | 0.48 | 0.47 |
| mixOmics | D | 0.5 | 0.6 | 0.55 | 1 | 0.67 | 0.8 | 0.6 | 0.75 | 0.67 | 0.67 | 0.67 |

Table S10: Precision, Recall, F_1_ scores for individual classes along with average F_1_ and accuracy scores for Small noisy datasets group

| Tool | Dataset | Class 1 | | | Class 2 | | | Class 3 | | | Average F_1_ | Accuracy |
| --- | --- | --- | --- | --- | --- | --- | --- | --- | --- | --- | --- | --- |
|  |  | Precision | Recall | F_1_ | Precision | Recall | F_1_ | Precision | Recall | F_1_ |  |  |
| SNF | A | 0.57 | 0.4 | 0.47 | 0.73 | 0.8 | 0.76 | 0.75 | 0.9 | 0.82 | 0.68 | 0.7 |
| SNF | B | 0.5 | 0.4 | 0.44 | 0.76 | 0.65 | 0.7 | 0.7 | 0.95 | 0.81 | 0.65 | 0.67 |
| SNF | C | 0.46 | 0.6 | 0.52 | 0.5 | 0.35 | 0.41 | 0.4 | 0.4 | 0.4 | 0.44 | 0.45 |
| SNF | D | 0.43 | 0.45 | 0.44 | 0.67 | 0.4 | 0.5 | 0.59 | 0.8 | 0.68 | 0.54 | 0.55 |
| BCC | A | 0.8 | 0.4 | 0.53 | 0.8 | 1 | 0.89 | 0.72 | 0.9 | 0.8 | 0.74 | 0.77 |
| BCC | B | 0.6 | 0.15 | 0.24 | 0.78 | 0.9 | 0.84 | 0.59 | 0.95 | 0.73 | 0.6 | 0.67 |
| BCC | C | 0.33 | 1 | 0.5 | 0 | 0 | 0 | 0 | 0 | 0 | 0.17 | 0.33 |
| BCC | D | 0.5 | 0.05 | 0.09 | 0.4 | 0.95 | 0.56 | 0.6 | 0.3 | 0.4 | 0.35 | 0.43 |
| iClusterPlus | A | 1 | 1 | 1 | 1 | 1 | 1 | 1 | 1 | 1 | 1 | 1 |
| iClusterPlus | B | 0.43 | 0.15 | 0.22 | 0.69 | 1 | 0.82 | 0.67 | 0.8 | 0.73 | 0.59 | 0.65 |
| iClusterPlus | C | 0.7 | 0.95 | 0.81 | 0.39 | 0.35 | 0.37 | 0.67 | 0.5 | 0.57 | 0.58 | 0.6 |
| iClusterPlus | D | 0.7 | 0.95 | 0.81 | 0.39 | 0.35 | 0.37 | 0.67 | 0.5 | 0.57 | 0.58 | 0.6 |
| mixOmics | A | 0.56 | 1 | 0.72 | 1 | 0.5 | 0.67 | 1 | 0.75 | 0.86 | 0.75 | 0.73 |
| mixOmics | B | 0.5 | 0.6 | 0.55 | 1 | 0.67 | 0.8 | 0.6 | 0.75 | 0.67 | 0.67 | 0.67 |
| mixOmics | C | 0.33 | 0.4 | 0.36 | 0.17 | 0.17 | 0.17 | 0.33 | 0.25 | 0.28 | 0.27 | 0.27 |
| mixOmics | D | 0.5 | 0.4 | 0.44 | 0.5 | 0.33 | 0.4 | 0.43 | 0.75 | 0.55 | 0.46 | 0.47 |

Table S11: Precision, Recall, F_1_ scores for individual classes along with average F_1_ and accuracy scores for Large datasets group

| Tool | Dataset | Class 1 | | | Class 2 | | | Class 3 | | | Average F_1_ | Accuracy |
| --- | --- | --- | --- | --- | --- | --- | --- | --- | --- | --- | --- | --- |
|  |  | Precision | Recall | F_1_ | Precision | Recall | F_1_ | Precision | Recall | F_1_ |  |  |
| SNF | A | 0.49 | 0.46 | 0.47 | 0.78 | 0.92 | 0.84 | 0.68 | 0.6 | 0.64 | 0.65 | 0.66 |
| SNF | B | 0.41 | 0.32 | 0.36 | 0.66 | 0.54 | 0.59 | 0.71 | 1 | 0.83 | 0.59 | 0.62 |
| SNF | C | 0.75 | 0.6 | 0.67 | 0.39 | 0.36 | 0.37 | 0.61 | 0.78 | 0.68 | 0.57 | 0.58 |
| SNF | D | 0.4 | 0.4 | 0.4 | 0.48 | 0.48 | 0.48 | 0.64 | 0.64 | 0.64 | 0.51 | 0.51 |
| BCC | A | 0.96 | 0.98 | 0.97 | 0.98 | 1 | 0.99 | 1 | 0.96 | 0.98 | 0.98 | 0.98 |
| BCC | B | NA | 0 | 0 | 0.62 | 1 | 0.77 | 0.69 | 0.96 | 0.8 | 0.52 | 0.65 |
| BCC | C | 0.6 | 0.98 | 0.74 | 0 | 0 | 0 | 0.68 | 0.94 | 0.79 | 0.51 | 0.64 |
| BCC | D | 0 | 0 | 0 | 0.33 | 1 | 0.5 | 0 | 0 | 0 | 0.17 | 0.33 |
| iClusterPlus | A | 1 | 1 | 1 | 1 | 1 | 1 | 1 | 1 | 1 | 1 | 1 |
| iClusterPlus | B | 1 | 1 | 1 | 1 | 1 | 1 | 1 | 1 | 1 | 1 | 1 |
| iClusterPlus | C | 0.64 | 0.96 | 0.77 | 0.45 | 0.2 | 0.28 | 0.7 | 0.74 | 0.72 | 0.59 | 0.63 |
| iClusterPlus | D | 0.5 | 0.52 | 0.51 | 1 | 0.48 | 0.65 | 0.68 | 1 | 0.81 | 0.66 | 0.67 |
| mixOmics | A | 1 | 1 | 1 | 1 | 1 | 1 | 1 | 1 | 1 | 1 | 1 |
| mixOmics | B | 1 | 0.75 | 0.86 | 0.92 | 1 | 0.96 | 0.88 | 1 | 0.94 | 0.92 | 0.92 |
| mixOmics | C | 0.73 | 0.92 | 0.81 | 0.43 | 0.25 | 0.32 | 0.69 | 0.79 | 0.74 | 0.62 | 0.66 |
| mixOmics | D | 0.67 | 0.17 | 0.27 | 0.56 | 0.75 | 0.64 | 0.68 | 0.93 | 0.79 | 0.57 | 0.63 |

Table S12: Precision, Recall, F_1_ scores for individual classes along with average F_1_ and accuracy scores for Large noisy datasets group

| Tool | Dataset | Class 1 | | | Class 2 | | | Class 3 | | | Average F_1_ | Accuracy |
| --- | --- | --- | --- | --- | --- | --- | --- | --- | --- | --- | --- | --- |
|  |  | Precision | Recall | F_1_ | Precision | Recall | F_1_ | Precision | Recall | F_1_ |  |  |
| SNF | A | 0.42 | 0.44 | 0.43 | 0.57 | 0.58 | 0.57 | 0.87 | 0.82 | 0.84 | 0.61 | 0.61 |
| SNF | B | 0.36 | 0.28 | 0.32 | 0.63 | 0.48 | 0.54 | 0.66 | 0.96 | 0.78 | 0.55 | 0.57 |
| SNF | C | 0.6 | 0.62 | 0.61 | 0.4 | 0.34 | 0.37 | 0.7 | 0.78 | 0.74 | 0.57 | 0.58 |
| SNF | D | 0.47 | 0.46 | 0.46 | 0.47 | 0.4 | 0.43 | 0.71 | 0.82 | 0.76 | 0.55 | 0.56 |
| BCC | A | 1 | 0.96 | 0.98 | 0.94 | 1 | 0.97 | 1 | 0.98 | 0.99 | 0.98 | 0.98 |
| BCC | B | 0 | 0 | 0 | 0.5 | 0.98 | 0.66 | 0.75 | 0.78 | 0.76 | 0.47 | 0.59 |
| BCC | C | 0.64 | 0.96 | 0.77 | NA | 0 | 0 | 0.65 | 0.98 | 0.78 | 0.52 | 0.65 |
| BCC | D | 0.53 | 0.32 | 0.4 | 1 | 0.02 | 0.04 | 0.42 | 1 | 0.59 | 0.34 | 0.45 |
| iClusterPlus | A | 1 | 1 | 1 | 1 | 1 | 1 | 1 | 1 | 1 | 1 | 1 |
| iClusterPlus | B | 1 | 0.98 | 0.99 | 0.98 | 1 | 0.99 | 1 | 1 | 1 | 0.99 | 0.99 |
| iClusterPlus | C | 0.65 | 0.96 | 0.78 | 0.34 | 0.28 | 0.31 | 0.69 | 0.48 | 0.57 | 0.55 | 0.57 |
| iClusterPlus | D | 0.5 | 0.52 | 0.51 | 1 | 0.48 | 0.65 | 0.68 | 1 | 0.81 | 0.66 | 0.67 |
| mixOmics | A | 1 | 1 | 1 | 1 | 1 | 1 | 1 | 1 | 1 | 1 | 1 |
| mixOmics | B | 0.78 | 0.58 | 0.67 | 0.85 | 0.92 | 0.88 | 0.81 | 0.93 | 0.87 | 0.81 | 0.82 |
| mixOmics | C | 0.75 | 0.75 | 0.75 | 0.42 | 0.42 | 0.42 | 0.64 | 0.64 | 0.64 | 0.6 | 0.61 |
| mixOmics | D | 0.67 | 0.33 | 0.44 | 0.69 | 0.92 | 0.79 | 0.75 | 0.86 | 0.8 | 0.68 | 0.71 |

**Runtime for multi-staged and meta-dimensional tools**

Table S13: Runtime in minutes for tools integrating copy number (CN) / methylation (ME) and gene expression (GE)

| **Tool** | **Mesothelioma dataset** | **Pancreatic cancer dataset** | **Colon cancer dataset** | **Melanoma dataset** |
| --- | --- | --- | --- | --- |
| CNAmet (CN + GE) | 54.52 | 61.29 | 56.77 | 77.49 |
| iGC | 0.17 | 0.26 | 0.40 | 0.52 |
| PLRS | 2.94 | 12.67 | 8.27 | 18.52 |
| Oncodrive-CIS | 45 | 157 | 290 | 567 |
| CNAmet (ME + GE) | 35.11 | 34.25 | 44.76 | 61.19 |
| MethylMix | 0.27 | 0.27 | 0.30 | 0.26 |

Table S14: Runtime in minutes for the meta-dimensional tools for real and simulated dataset analyses

| **Dataset/Tool** | **SNF** | **BCC** | **iClusterPlus** | **mixOmics** |
| --- | --- | --- | --- | --- |
| HPC – Binary class classification | 0.011 | 26.57 | 1904.42 | 89.32 |
| HPC – Multi-class classification | 0.012 | 39.95 | 2495.80 | 145.12 |
| GBM – Multi-class classification | 0.010 | 15.00 | 1998.53 | 132.25 |
| Small datasets group – Simulated dataset A | 0.001 | 1.44 | 19.84 | 24.98 |
| Small noisy datasets group – Simulated dataset A | 0.001 | 1.53 | 21.45 | 27.06 |
| Small datasets group – Simulated dataset B | 0.001 | 1.38 | 19.92 | 20.5 |
| Small noisy datasets group – Simulated dataset B | 0.001 | 1.49 | 21.6 | 18.81 |
| Small datasets group – Simulated dataset C | 0.001 | 1.38 | 19.75 | 31.77 |
| Small noisy dataset group – Simulated dataset C | 0.001 | 1.46 | 21.32 | 30.46 |
| Small datasets group – Simulated dataset D | 0.001 | 1.4 | 19.95 | 30.7 |
| Small noisy datasets group – Simulated dataset D | 0.001 | 1.51 | 21.58 | 69.74 |
| Large datasets group – Simulated dataset A | 0.008 | 1.56 | 49.29 | 24 |
| Large noisy datasets group – Simulated dataset A | 0.008 | 1.68 | 53.33 | 23.19 |
| Large datasets group – Simulated dataset B | 0.006 | 1.61 | 49.29 | 22 |
| Large noisy datasets group – Simulated dataset B | 0.006 | 1.66 | 53.45 | 26.5 |
| Large datasets group – Simulated dataset C | 0.006 | 1.5 | 48.72 | 33.54 |
| Large noisy datasets group – Simulated dataset C | 0.006 | 1.63 | 52.99 | 24.91 |
| Large datasets group – Simulated dataset D | 0.006 | 1.49 | 49.58 | 34.16 |
| Large noisy datasets group – Simulated dataset D | 0.006 | 1.68 | 53.64 | 33.79 |
| **Average Time (minutes)** | **0.005** | **5.57** | **367.08** | **44.36** |

**References**

1. Leday GG, van der Vaart AW, van Wieringen WN et al. Modeling association between DNA copy number and gene expression with constrained piecewise linear regression splines. *Ann Appl Stat* 2013;**7**(2):823-45.

2. Tamborero D, Lopez-Bigas N, Gonzalez-Perez A. Oncodrive-CIS: a method to reveal likely driver genes based on the impact of their copy number changes on expression. *PLoS One* 2013;**8**(2):e55489.

3. Gevaert O, Tibshirani R, Plevritis SK. Pancancer analysis of DNA methylation-driven genes using MethylMix. *Genome Biol* 2015;**16**:17.

4. Shen R, Olshen AB, Ladanyi M. Integrative clustering of multiple genomic data types using a joint latent variable model with application to breast and lung cancer subtype analysis. *Bioinformatics* 2009;**25**(22):2906-12.

5. Curtis C, Shah SP, Chin S-F et al. The genomic and transcriptomic architecture of 2,000 breast tumours reveals novel subgroups. *Nature* 2012;**486**(7403):346-52.

6. Network CGAR. Comprehensive molecular profiling of lung adenocarcinoma. *Nature* 2014;**511**(7511):543.

7. Network CGAR. Integrated genomic characterization of oesophageal carcinoma. *Nature* 2017;**541**(7636):169.

8. Singh A, Gautier B, Shannon CP et al. DIABLO: from multi-omics assays to biomarker discovery, an integrative approach. *bioRxiv* 2018:067611.
